# Leucine-Rich Repeat Kinase 2 Controls the Ca^2+^/Nuclear Factor of Activated T Cells/IL-2 Pathway during *Aspergillus* Non-Canonical Autophagy in Dendritic Cells

**DOI:** 10.3389/fimmu.2018.00210

**Published:** 2018-02-08

**Authors:** Alicia Yoke Wei Wong, Vasilis Oikonomou, Giuseppe Paolicelli, Antonella De Luca, Marilena Pariano, Jan Fric, Hock Soon Tay, Paola Ricciardi-Castagnoli, Teresa Zelante

**Affiliations:** ^1^Singapore Immunology Network, Agency for Science, Technology and Research, Singapore, Singapore; ^2^National University of Singapore Graduate School for Integrative Sciences and Engineering, National University of Singapore, Singapore, Singapore; ^3^Department of Experimental Medicine, University of Perugia, Perugia, Italy; ^4^Center for Translational Medicine (CTM), International Clinical Research Center (ICRC), St. Anne’s University Hospital Brno, Brno, Czechia; ^5^Toscana Life Sciences Foundation, Siena, Italy

**Keywords:** leucine-rich repeat kinase 2, nuclear factor of activated T cells, NRON, dendritic cell, *Aspergillus*, autophagy

## Abstract

The Parkinson’s disease-associated protein, Leucine-rich repeat kinase 2 (LRRK2), a known negative regulator of nuclear factor of activated T cells (NFAT), is expressed in myeloid cells such as macrophages and dendritic cells (DCs) and is involved in the host immune response against pathogens. Since, the Ca^2+^/NFAT/IL-2 axis has been previously found to regulate DC response to the fungus *Aspergillus*, we have investigated the role played by the kinase LRRK2 during fungal infection. Mechanistically, we found that in the early stages of the non-canonical autophagic response of DCs to the germinated spores of *Aspergillus*, LRRK2 undergoes progressive degradation and regulates NFAT translocation from the cytoplasm to the nucleus. Our results shed new light on the complexity of the Ca^2+^/NFAT/IL-2 pathway, where LRRK2 plays a role in controlling the immune response of DCs to *Aspergillus*.

## Introduction

Leucine-rich repeat kinase 2 (LRRK2) was first discovered in genome-wide linkage studies of patients of Parkinson’s disease (1, 2). Since then, the majority of studies on LRRK2 have been focused on linking various point mutations in the various domains of LRRK2 with Parkinson’s disease (3), and the contribution of mutated LRRK2 protein to neuronal toxicity (4–6). Studies have also shown a possible involvement of the immune system in Parkinson’s disease pathogenesis. Inflammation is thought to lead to the neurodegeneration and neurotoxicity seen in Parkinson’s disease (7, 8). Also, it is known that patients of Parkinson’s disease are often hospitalized for various types of infections, such as urinary tract infection and pneumonia (9). The involvement of LRRK2 in response to inflammatory stimulus (10), as well as microbes, and pathogen-associated molecular patterns (PAMPs) (11–14), have also been seen in several studies.

Leucine-rich repeat kinase 2 has been reported to negatively regulate the nuclear factor of activated T cells (NFAT) pathway in bone marrow-derived macrophages (BMDMs) *via* the NRON complex. LRRK2 was found to bind 5 of the 11 proteins associated with the NRON complex, and overexpression of LRRK2 increases binding of NFAT with NRON complex members, in particular IQGAP, chromosome segregation 1-like (CSE1L), and transportin-1 (15). This regulation of NFAT by LRRK2 was found to be independent of its kinase function. Given the importance of the NFAT pathway in the regulation of T cell development and function [as reviewed in Ref. (16)], it is of notice that LRRK2 expression is lower in T cells than in BMDMs and bone marrow-derived dendritic cells (BMDCs) (15), underlining a possible role of LRRK2 in innate immunity during infection as supported by other studies (10, 17).

It has been established that the Ca^2+^/NFAT/IL-2 pathway is activated in DCs in response to live *Candida albicans* and zymosan binding to Dectin-1 leading to the production of cytokines, including IL-2 (18). To this end, we have also shown that NFAT signaling regulates cytokine IL-2 expression in DCs stimulated *in vitro* with *Saccharomyces cereviseae*-derived whole glucan particles (19), and that DC-derived IL-2 expression in lung CD103^+^ DCs is important for eliciting the appropriate Th17 cell response to *Aspergillus fumigatus* infection (20). In this study, we report that LRRK2 localizes to lysosomic and endosomic structures in DCs at steady state, as well as after *Aspergillus* exposure, and that autophagy is able to influence LRRK2 expression, and subsequently, the activation of Ca^2+^/NFAT/IL-2 axis in DCs differently from the other NRON complex components. In conclusion, our findings suggest a role for LRRK2 and the NRON complex in the early phases of the immune response to *Aspergillus*.

## Materials and Methods

### Mice

Eight-week-old wild-type C57BL/6 mice used for experiments were bred and kept under specific pathogen-free conditions in the Biomedical Resource Centre, Singapore. All experiments and procedures were approved by the Institutional Animal Care and Use Committee (IACUC) of A*STAR (Biopolis, Singapore) (Authorization No.: IACUC 110626) in accordance with the guidelines of the Agri-Food and Veterinary Authority (AVA) and the National Advisory Committee for Laboratory Animal Research (NACLAR) of Singapore. Z. Liu of Institute of Biophysics Chinese Academy of Sciences provided LRRK2^−/−^ mice bone marrows.

### Fungal Cells

*Aspergillus fumigatus* isolate AF293 (MYA-4609, ATCC, Manassas, VA, USA) was used for cultures. *A. fumigatus* was cultivated for 5 days on potato dextrose agar (Sigma-Aldrich, St. Louis, MO, USA) before conidia were harvested by washing with PBS 0.05% Tween 20. Swollen conidia were obtained by incubating conidia in liquid yeast extract peptone dextrose at 37°C for 4 h on a rotary shaker at 300 rpm. In addition, a red fluorescent protein (RFP)-expressing *A. fumigatus* AF293 strain used in this study was obtained from Prof. Eric Pearlman (Case Western Reserve University, Cleaveland, OH, USA).

### Cell Cultures

Bone marrow-derived dendritic cells were generated by culturing mouse bone marrow extracted by flushing the femurs and tibias. The collected bone marrow was treated with ammonium-chloride-potassium lysing buffer. The remaining cells post-lysis were cultured in suspension plates with Iscove’s Modified Dulbeccos Medium (IMDM) (HyClone, Thermo Fisher Scientific, Waltham, MA, USA) containing 10% fetal bovine serum (FBS) (Euroclone, Milan, Italy), 2 mM L-glutamine (Gibco, Life Technologies, Carlsbad, CA, USA), 100 U/mL Penicillin/Streptomycin (Gibco, Life Technologies, Carlsbad, CA, USA), supplemented with 10% supernatant from a GM-CSF-producing B16 melanoma cell line to a final concentration of 20 ng/mL GM-CSF to generate BMDCs.

The long-term GM-CSF cytokine-dependent DC cell line, D1 (21), was grown in suspension plates with IMDM containing 10% FBS (Australian origin, Gibco, Life Technologies, Carlsbad, CA, USA) 2 mM L-glutamine, 100 U/mL Penicillin/Streptomycin, and 55 µM β-mercaptoethanol (Gibco, Life Technologies, Carlsbad, CA, USA), supplemented with 30% supernatant from NIH/3T3 cells transfected to produce GM-CSF to a final concentration of 10 ng/mL.

In all stimulation experiments, cells were stimulated with *A. fumigatus* swollen conidia in a 1:10 (fungi:cell) ratio. Pharmaceuticals used in these experiments include 3-methyladenine (3MA) (Sigma-Aldrich, St. Louis, MO, USA), Bafilomycin A (Baf) from *Streptomyces griseus* (Calbiochem, Merck Millipore, Billerica, MA, USA), Cytochalasin D (CytoD) (Sigma-Aldrich, St. Louis, MO, USA), leupeptin hemisulfate (Sigma-Aldrich, St. Louis, MO, USA), and ammonium chloride (Leu/A) (Sigma-Aldrich, St. Louis, MO, USA).

### NFAT Nuclear Translocation

D1 nuclear factor of activated T cells translocation-firefly luciferase reporter cells were generated by transducing the D1 cells with Cignal Lenti NFAT Reporter with firefly luciferase (SABiosciences, Qiagen, Venlo, Limburg, Netherlands). NFAT nuclear translocation was detected by ONE-Glo™ Luciferase assay System (Promega, Madison, WI, USA) and the luminescence signal quantified with the GloMax^®^-Multi Detection System Luminometer module (Promega, Madison, WI, USA).

### shRNA Knockdown

MISSION^®^ Lentiviral Particles (Sigma-Aldrich, St. Louis, MO, USA) with the pLKO.1-puro vector containing shRNA sequences targeting NRON, CSE1L, sperm-associated antigen 9 (SPAG9), LRRK2 shRNA, PPP2R1A were used.

Below details includes the gene reference identification number (NM ID) and The RNAi Consortium (TRC) clone identification number (clone ID) for the shRNA-lentiviral particles for targeting these genes (Table 1).

**Table 1:** Details of commercially purchased MISSION^®^ lentiviral particles.

Gene target	NM ID	The RNAi Consortium (TRC) clone ID
Chromosome segregation 1-like	NM_023565	TRCN0000174506 TRCN0000174691
Sperm-associated antigen 9	NM_027569	TRCN0000176696 TRCN0000177089
*Ppp2r1a*	NM_016891	TRCN0000012624 TRCN0000012626
Leucine-rich repeat kinase 2	NM_025730	TRCN0000022656 TRCN0000022657

NRON shRNA lentiviral particles were custom designed and packaged into lentiviral particles (ACGGTGGGTTTATGACAAATT and ACGGGTGCTGGATGACATATT) by Sigma-Aldrich (St. Louis, MO, USA). MISSION^®^ pLKO.1-puro non-target shRNA control transduction particles (Sigma-Aldrich, St. Louis, MO, USA) were used as a transduction control.

For the transduction, D1 cells were seeded and rested overnight in antibiotic-free D1 medium. The next day, the medium was replaced with antibiotic-free D1 medium containing 2 µg/mL SureEntry™ transduction reagent (SABiosciences, Qiagen, Venlo, Limburg, Netherlands). The D1 cells were then transduced with MISSION^®^ lentiviral particles at multiplicity of infection (MOI) 10 and allowed to incubate for 20 h at 37°C in 5% CO_2_. After 20 h incubation, the medium containing the lentivirus particles was aspirated out, replaced with complete D1 medium, and allowed to rest for 48 h at 37°C in 5% CO_2_. After being allowed to rest, successfully transduced cells were selected 4 days by replacing the medium with D1 medium containing 0.5 µg/mL of puromycin dihydrochloride (Calbiochem, Merck Millipore, Billerica, MA, USA).

For *in vitro* silencing of *Rubcn, Nox2*, and *Atg7*, D1 were transfected with 40 nM of the following siRNAs: *Rubcn* (Duplex name mm.Ri.1700021K19Rik.13.1; sense, 5′-GUACUUGACCGCUAGUAAAAUCATT-3′; antisense, 5′-GACAUGAACUGGCGAUCAUUUUAGUAA-3′), Nox2 (Duplex name mm.Ri.Cybb.13.1; sense, 5′-GUUCAAGGUCAGUUUAUUGAAUGAA-3′; antisense, 5′-CACAAGUUCCAGUCAAAUAACUUACUU-3’), Atg7 (Duplex name mm.Ri.Atg7.13.1; sense, 5′CUUGAUCAGUACGAGCGAGAAGGAT-3′; antisense, 5′-AAGAACUAGUCAUGCUCGCUCUUCCUA-3′) (all from IDT). Silencing was performed using TransIT-TKO^®^ Transfection Reagent (Mirus) and incubated for 24 h (as indicated by preliminary experiments performed at 12, 24, or 48 h) at 37°C in 5% CO_2_. Transfected cells were exposed to A-sw.

### Cytotoxicity Assay

The sensitivity of cells to different inhibitors was determined by 3-(4,5-dimethylthiazol-2-yl)-2,5-diphenyltetrazolium bromide (MTT; Trevigen) assay. Briefly, 2,500 D1 cells were seeded in 180 µL medium in quadruplicate in 96-well tissue culture plates and following overnight incubation with A-sw in a 1:10 (fungi:cell) ratio with or without inhibitors. After 24 h MTT was added and the absorbance of the formazan product was measured on a microplate reader TECAN infinite m200 together with the i-control™ software (Mannedorf, Switzerland) at 600 nm.

### Lysosome Isolation

Lysosomes were isolated from D1 cells as described by Graham (22). Briefly, D1 cells were harvested from suspension plates and washed with PBS. The cell pellet was then resuspended in ice cold homogenization medium (0.25 M sucrose, 1 mM EDTA, 10 mM HEPES, pH 7) and homogenized in a Wheaton type Dounce tissue grinder (Wheaton, Millville, NJ, USA) on ice until above 90% cell breakage was observed under the microscope by staining with PBS containing 0.04% v/v Tryphan blue (Sigma-Aldrich, St. Louis, MO, USA). The homogenate was then centrifuged at 800 *g* for 10 min to pellet nuclei and cell debris. The resulting supernatant from the centrifuge was mixed with bovine serum albumin (final proportion of 4% v/v) and Percoll (final proportion of 22% v/v). The mixture was then ultracentrifuged for 30 min at 36,000 × *g* without brake activation. After centrifugation, a visible band of the enriched lysosomes was seen near the bottom of the tube. 400 µL fractions, containing the enriched lysosomes were collected, and Igepal CA-630 (final proportion of 0.5% v/v) was used to solubilize the lysosome membranes. Solubilized fractions were centrifuged at 100,000 × *g* for 2 h to pellet the Percoll, and the resulting supernatants were obtained for analysis by western blotting.

### Cytokine Detection

Cell culture medium was assayed for cytokine production by sandwich enzyme-linked immunosorbent assay (ELISA) for IL-2, IL-12/IL-23p40, and IL-23. IL-2 and IL-12/IL-23p40 were assayed using commercially available antibody pairs and standards from BioLegend (San Diego, CA, USA) and eBioscience (San Diego, CA, USA), respectively. Measurements were detected using the TECAN infinite m200 together with the i-control™ software (Mannedorf, Switzerland; measurement wavelength 450 nm, reference wavelength 570 nm). IL-23 was assayed using mouse IL-23 ELISA Ready-SET-Go!^®^ (Second-generation assay) (Affymetric eBioscience, San Diego, CA, USA) according to manufacturer’s instructions. In addition, selected supernatant samples were analyzed using the Milliplex Multi Analyte Panels Mouse TH17 Magnetic Bead Panel Immunology Multiplex assay (MTH17MAG-47K) in conjunction with the Luminex MAGPIX^®^ system (Merck Millipore, Billerica, MA, USA).

### Western Blot

Whole cell lysates were obtained by lysing cells in radioimmunoprecipitation assay buffer containing 1× cOmplete, EDTA-free Protease Inhibitor Cocktail (Roche, Basel, Switzerland), 1× PhosSTOP Phosphatase Inhibitor Cocktail (Roche, Basel, Switzerland), and 1 mM phenylmethanesulfonylfluoride. The protein concentration in the cell lysates were measured using the Pierce™ 660 nm Protein assay kit (Thermo Scientific, Waltham, MA, USA), and colorimetric readings were obtained by measuring the wavelength at 660 nm using the TECAN infinite m200 together with the i-control™ software (Mannedorf, Switzerland). Protein lysates were diluted with Laemmli buffer containing 2.5% v/v β-mercaptoethanol (Sigma-Aldrich, St. Louis, MO, USA) and heated at 95°C for 10 min prior to separation on sodium dodecyl sulfate (SDS)-polyacrylamide gel electrophoresis (PAGE) in Tris-glycine-SDS buffer, along side the Precision Plus Protein Dual Color standards protein ladder (Bio-Rad, Hercules, CA, USA). Up to 50 µg of protein was loaded into each well for separation. The SDS-PAGE gel was run at 60 V till all samples entered the resolving gel, before increasing the running voltage to 110 V until the dye front had traveled to the end of the gel. The separated proteins were then transferred to polyvinylidene fluoride membrane in Tris-glycine buffer containing 10% v/v methanol at either 240 mA for 2 h or 200 mA for 4 h. For LRRK2, lysates were first concentrated using the Vivaspin 500 molecular weight cut off 100,000 columns (Sartorius, Goettingen, Germany), and the NuPAGE^®^ large protein analysis system (Life Technologies, Carlsbad, CA, USA) was adopted. Antibodies used for western blot include: LRRK2 rabbit monoclonal antibody, clone: MJFF2 (Epitomics, Abcam, Cambridge, UK), LC3B antibody (cell Signaling Technology, Danvers, MA, USA), Purified anti-mouse CD107a (LAMP-1), clone: 1D4B (Biolegend, San Diego, CA, USA), anti-TATA binding protein (TBP), clone: 1TBP18 (Abcam, Cambridge, UK), Rab5 (C8B1) Rabbit mAb (cell Signaling Technology, Danvers, MA, USA), JIP4/SPAG9 (D72F4) XP^®^ Rabbit mAb (cell Signaling Technology, Danvers, MA, USA), Anti-PPP2R1A antibody [6F9] (Abcam, Cambridge, UK), GAPDH antibody, clone: 6C5 (Merck Millipore, Billerica, MA, USA), DAPK1 mouse polyclonal antibody, clone: RB3033 (antibodies-online.com). For DAPK1, normalization was performed by probing the membrane with mouse monoclonal β-actin antibody, clone: AC-40 (Sigma-Aldrich). Band pixel density was analyzed from the film scans by ImageJ software (NIH, Bethesda, MD, USA).

### RNA Extraction, Real-time Quantitative PCR

Cells were lysed with 1 mL of TRIzol^®^ reagent (Ambion, Life Technologies, Carlsbad, CA, USA) and flash frozen on dry ice prior to storage at −80°C. To extract RNA, 200 µL (20% v/v) of chloroform (Merck, Kenilworth, NJ, USA) was added to each sample. Each sample was then shaken vigorously, and then centrifuged at 15,000 rpm, 4°C for 15 min. This separated the sample into a top organic phase containing the RNA, a thin interface containing DNA and a bottom organic phase containing protein. The top supernatant was aspirated and transferred to a new tube. An equal volume of 70% v/v ethanol was added and mixed to the sample. RNA was extracted from this mixture using the RNeasy mini kit (Qiagen, Venlo, Limburg, Netherlands) according to the manufacturer’s instructions. Extracted RNA samples were then treated with DNase using the TURBO DNA-free™ kit (Ambion, Life Technologies, Carlsbad, CA, USA) according to manufacturer’s instructions. The RNA content of each sample was then quantified using the Nanodrop™ 1000 (Thermo Scientific, Waltham, MA, USA). 2 µg of RNA was retro-transcribed into cDNA using the High Capacity Reverse Transcription Kit (Applied Biosystems, Life Technologies, Carlsbad, CA, USA) according to manufacturer’s instruction. The obtained cDNA was then used for real-time quantitative PCR. The Brilliant SYBR^®^ Green QPCR Mastermix and Reference Dye (Stratagene, Agilent Technologies, Santa Clara, CA, USA) was used to set up the real-time quantitative PCR reaction, and the samples were run in the MX3000P instrument (Stratagene, Agilent Technologies, Santa Clara, CA, USA). Gene expression data was analyzed using the MxPro QPCR software (Stratagene, Agilent Technologies, Santa Clara, CA, USA). Primer sequences targeting mouse genes used in this study are listed in Table 2.

**Table 2:** List of real-time quantitative PCR primers.

Gene	Forward primer (5′–3′)	Reverse primer (5′–3′)
Chromosome segregation 1-like	GTGGGAAAGGACAGGAAACA	AAACCTTGGTGATCGTTTGC
*Gapdh*	TTGAGGTCAATGAAGGGGTC	TCGTCCCGTAGACAAAATGG
Leucine-rich repeat kinase 2	GATGTCAGTACGCCCCTGAT	CTGCCAGCGCTATGATGTTA
*Nron*	CAGTAAAGGAGCAGTAGTGGAAACAG	TGGGGGGAGCGAATGGCATCGGGAAC
*Ppp2r1a*	CAACCTGGATTGGTGGAACG	GATCCACTAGCCAGGCCATA
Sperm-associated antigen 9	AAACCTCAGGGACTCCAGGT	CCCCACCACTGCTACTTTGT
*Rubcn*	GUACUUGACCGCUAGUAAAAUCATT	GACAUGAACUGGCGAUCAUUUUAGUAA
*Atg7*	CUUGAUCAGUACGAGCGAGAAGGAT	AAGAACUAGUCAUGCUCGCUCUUCCUA
*Nox2*	GUUCAAGGUCAGUUUAUUGAAUGAA	CACAAGUUCCAGUCAAAUAACUUACUU
*Dapk1*	CGAGUUUGGAUAUGACAAGGAUACA	UGUAUCCUUGUCAUAUCCAAACUCGCC

### Immunofluorescence Confocal Microscopy

D1 cells were seeded and stimulated in μ-slide 8-well chambers (Ibidi, Martinsried, Germany). Post-stimulation cells were fixed in 2% paraformaldehyde (Sigma-Aldrich, St. Louis, MO, USA), and permeabilized in PBS containing 0.1% saponin (Sigma-Aldrich, St. Louis, MO, USA), 0.2% gelatin (Fluka Analytical, Sigma-Aldrich, St. Louis, MO, USA), and 5% bovine serum albumin (Merck Millipore, Darmstadt, Germany). Cells were stained and washed using PBS containing 0.01% saponin and 0.2% gelatin. Cells were stained with a primary LC3B antibody (Cell Signaling Technology, Danvers, MA, USA) and a secondary goat α-rabbit IgG antibody conjugated to Alexa Fluor^®^ 488 (Invitrogen, Life Technologies, Carlsbad, CA, USA). In addition, 4’,6-diamidino-2-phenylindole (Invitrogen, Life Technologies, Carlsbad, CA, USA) was used to stain the cell nuclei. Images were acquired on an Olympus FV1000 confocal microscope in conjunction with the Fluorview FV1000 software (Olympus, Tokyo, Japan).

For DAPK1 staining, DAPK1 mouse polyclonal antibody, clone: RB3033 (antibodies-online.com) and Alexa Fluor^®^ 488 phalloidin was used for selective labeling of F-actin. After overnight staining with primary antibody, slides were washed and incubated with Rabbit IgG-TRITC antibody (Sigma-Aldrich). Images were acquired using a Zeiss Axio Observer. Z1 inverted microscope, equipped with Apotome filter and Axiocam MRm camera detection system Zeiss using a 63×/1.25 oil Plan/neofluar objective.

### Transmission Electron Microscopy

Stimulated D1 cells were harvested from the suspension plate with 2 mM EDTA in PBS and washed with 0.2 M sodium cacodylate buffer (pH 7.4). Cells were then fixed in cacodylate fixative buffer (0.1 M sodium cacodylate, 2% paraformaldehyde, and 3% gluteraldehyde) overnight at 4°C. The cells were then washed with 0.2 M sodium cacodylate buffer and dehydrated on an alcohol series (30, 50, 70, 80, 90, and 100%) for 15 min each. Specimens were then embedded into acrylic resin. Ultrafine sections were obtained by cutting into the resin specimens with a glass blade on an ultramicrotome, and mounted on nickel grids. The grids were then washed with PBS and then stained with antibodies LRRK2 rabbit monoclonal antibody clone: MJFF2 (Epitomics, Abcam, Cambridge, UK) and purified anti-mouse CD107a (LAMP-1) clone: 1D4B (Biolegend, San Diego, CA, USA), followed by secondary antibodies that have been conjugated with either 5 or 15 nm gold particles (Cytodiagnostics, Burlington, ON, Canada). All antibody incubations were done in PBS containing 1% bovine serum albumin. After antibody staining, grids were post-fixed with cacodylate fixative buffer for 15 min, and then stained with 2% uranyl acetate. Micrographs were taken with an EM 208 transmission electron microscope (Phillips, Amsterdam, Netherlands).

### Graphs and Statistics

Statistical significance of experiments was determined either by Student’s *t*-test, one-way ANOVA, or two-way ANOVA as indicated. For statistics generated by one-way ANOVA, the differences between individual groups were compared using the Bonferroni’s Multiple Comparison post-test. All graphs and statistics were generated using the Graphpad Prism^®^ software version 6.0 (Graphpad Software, La Jolla, CA, USA).

### Flow Cytometry Analysis

At 16 h post-silencing, BMDCs were treated with *Aspergillus* RFP swollen conidia (ratio of 1:1) for 1 h. BMDCs were collected, washed twice with FACS buffer, stained with CD11c-Alexa Fluor 700^®^ (BD) and analyzed by flow cytometry. Data (mean ± SD) represent three independent experiments in which technical triplicates of 100,000 cells per sample were acquired using a Fortessa cytometer (BD). Phagocytosis was calculated using flow cytometry analysis (described above). The percentage of phagocytosis equals the number of BMDCs that have engulfed RFP *A. fumigatus*.

### Time Lapse Video

Bone marrow-derived dendritic cells grown in 48-well plates were exposed with shRNA RUBICON or scramble overnight. Cells were then treated with *Aspergillus* swollen conidia for 1 h. Time-lapse imaging was performed over a period of 1 h with a 40× objective using the time-lapse function of the EVOS FL Auto Imaging System with the Invitrogen™ EVOS™ Onstage Incubator.

## Results

### LRRK2 Is Expressed in DCs and Is Regulated during *A. fumigatus* Infection

**Figure 1:**
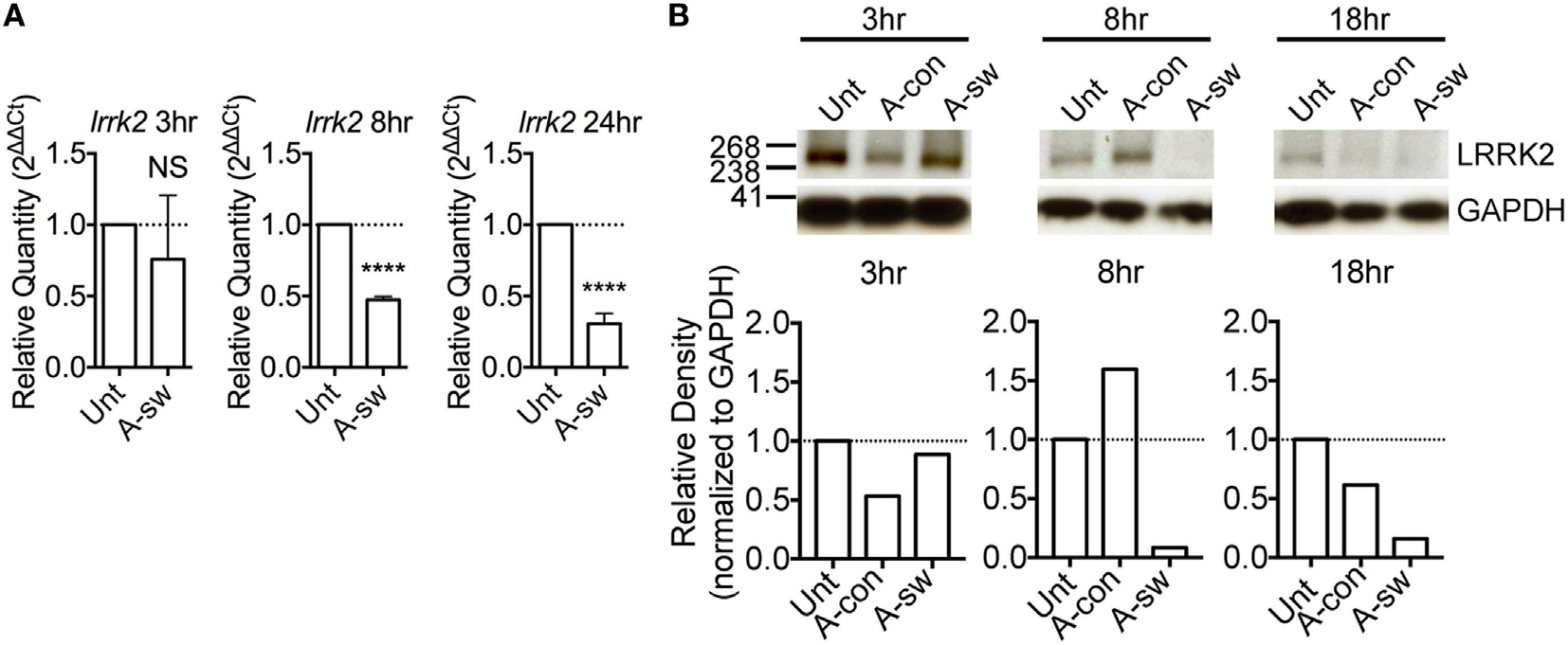
Leucine-rich repeat kinase 2 (LRRK2) is expressed in dendritic cells and is downregulated by *Aspergillus fumigatus*. **(A)** LRRK2 mRNA in D1 cells. Data is displayed as the mean gene expression ± SD of two biological replicates and normalized to the housekeeping protein, GAPDH. Differences found to be statistically significant by one-way ANOVA with Bonferonni’s Multiple Comparison post-test are indicated (NS, non-significant; ****, *p* < 0.0001). **(B)** Protein expression level of LRRK2 in whole cell lysates of D1 cells stimulated with *A. fumigatus* condia and swollen conidia. For densitometry analysis, band densities for LRRK2 were normalized to the band density of the housekeeping protein, GAPDH, after which the density of the LRRK2 band of the treated sample was compared with that of the untreated sample. Data is representative of three independent experiments. Abbreviations used: Untreated (Unt); *A. fumigatus* conidia (A-con); *A. fumigatus swollen conidia* (A-sw).

Leucine-rich repeat kinase 2 is known to regulate NFAT translocation in response to TLR ligands (15). Since, we have previously shown that *A. fumigatus* swollen conidia, more than ungerminated conidia, induces Ca^2+^/NFAT/IL-2 pathway in DCs (20), here we went to study the regulation of LRRK2 in response to fungal infection. First, we measured LRRK2 expression at the mRNA and protein level in D1 cells at resting state as well as in D1 cells stimulated with swollen conidia. We found that at resting state, DCs express LRRK2 on both the mRNA (Figure 1A) and the protein level (Figure 1B). Upon stimulation with swollen conidia, LRRK2 levels were found to be downregulated on both the mRNA as well as the protein level. At 8 h post-stimulation, downregulation of LRRK2 on the gene level was found to be statistically significant, while LRRK2 protein levels decreased further significantly (8 h 0.217 ± 0.1). The downregulation of LRRK2 was further maintained on protein level at 18 h (0.373 ± 0.2), and on gene expression at 24 h post-stimulation, showing that *Aspergillus* is able to influence *Lrrk2* gene expression on both transcriptional and translational level. Interestingly, the downregulation of LRRK2 in DCs responding to *Aspergillus* swollen conidia corresponded to the upregulation of NFAT1 translocation (Figure S1A in Supplementary Material) and IL-2 production (Figure S1B in Supplementary Material).

### LRRK2 Intracellular Localization

**Figure 2:**
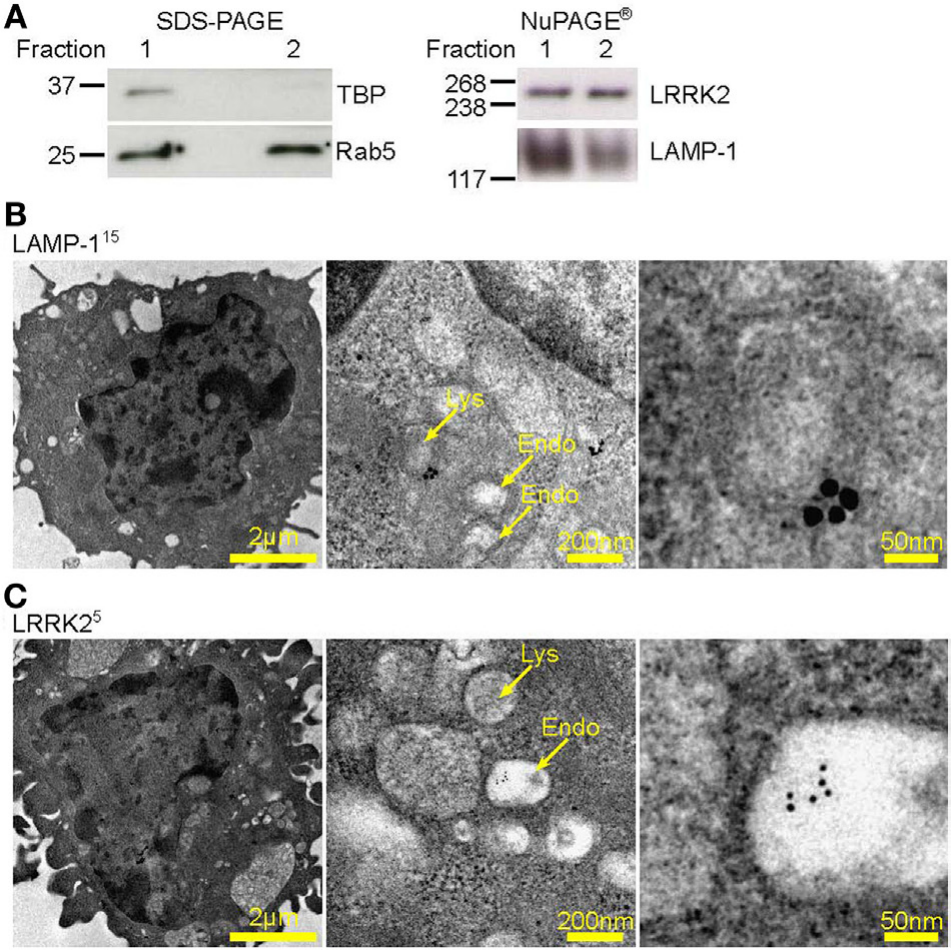
Leucine-rich repeat kinase 2 (LRRK2) is localized on lysosomes and endosomes of dendritic cells. **(A)** Western blot analysis of presence of TATA binding protein, Rab5, LRRK2, and LAMP-1 in a representative protein fraction obtained from the enrichment of lysosomes from D1 cells. Lysate from the protein fraction was run on both sodium dodecyl sulfate-polyacrylamide gel electrophoresis (PAGE) and NuPAGE^®^ to probe for the proteins indicated. Data are representative of three independent experiments **(B–C)**, D1 cells were processed for immune-electron microscopy, stained for LAMP-1 or LRRK2, and labeled with appropriate secondary antibodies conjugated with 5 or 15 nm gold particles (size of particles indicated in superscript). Lysosomic and endosomic structures are as indicated. Electron micrographs of D1 cells show LAMP-1 positive lysosome **(B)**, as well as endosomic structures positive for LRRK2 **(C)**. Size of scale bars are as indicated.

It has been reported that LRRK2 is degraded in lysosomes (23). In order to understand the subcellular localization of LRRK2 in DCs, lysosomes and early endosomes were enriched from D1 cells by gradient centrifugation protocol (22), and the protein content of the fractions enriched with these organelles were analyzed by western blot (Figure 2A). Fraction (lane 1) was harvested and further centrifuged (lane 2), which was negative for the nuclear marker, TBP, indicating that there was no contamination with nuclear material. We found that LRRK2 was present in fractions that expressed lysosomal-associated membrane protein 1 (LAMP-1) and Rab5, which are markers for lysosomes and early endosomes, respectively. This is in line with what was already reported (24, 25). In addition, by electron microscopy, lysosomes were LAMP-1 positive, spherical, enclosed by one membrane, with a diameter of 70–150 nm, homogenous and electron-dense interior. Endosomes were LAMP-1 negative and poorly electron-dense (Figure 2B). Interestingly, LRRK2 (Figure 2C) was found localized also on endosomic structures. In addition, as endosomes have been proposed to be signaling hubs, where components of signaling pathways can localize and interact (26), the localization of LRRK2 on endosomes implies that the endosomes serve as a possible niche were NFAT signaling pathway is kept under control.

### *A. fumigatus* Swollen Conidia Activate the Non-Canonical Autophagic Pathway in DCs

**Figure 3:**
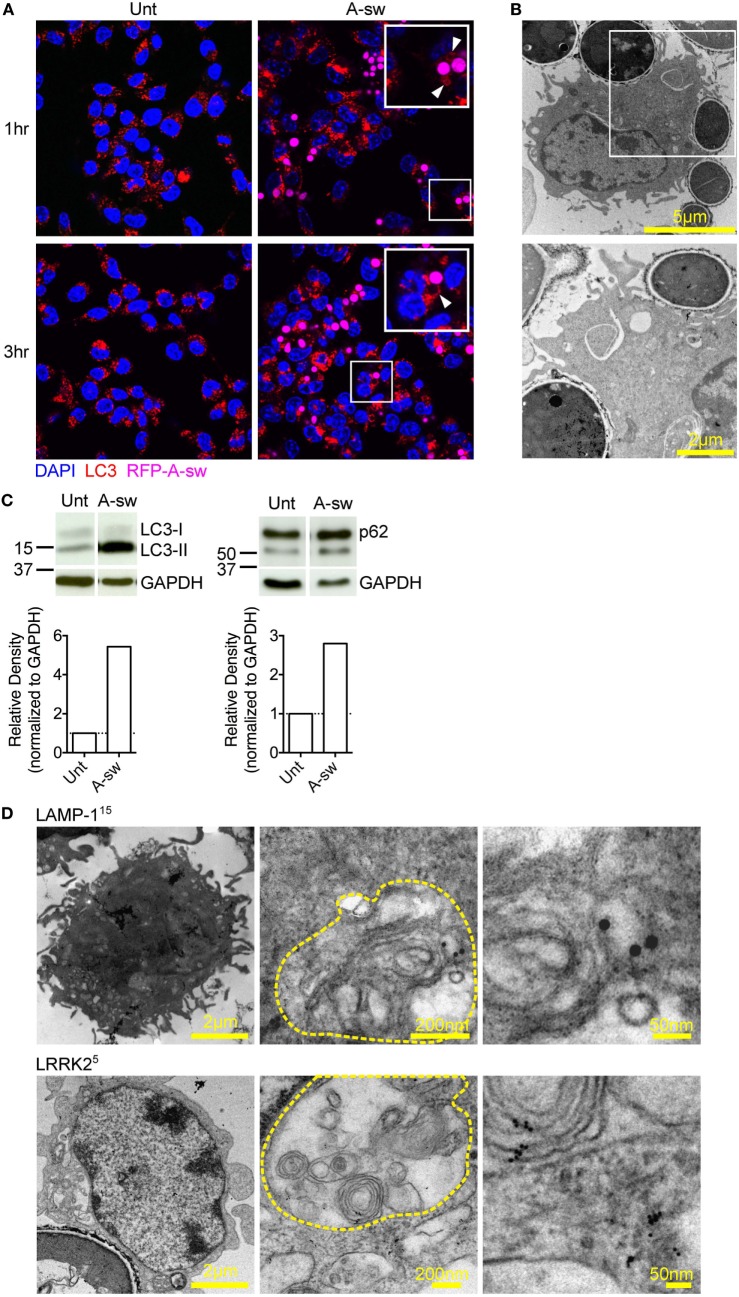
*Aspergillus fumigatus* swollen conidia activate non-canonical autophagy and the formation of multivesicular bodies in dendritic cells. D1 cells were stimulated with *A. fumigatus* swollen conidia for 3 h. **(A)** Immunofluorescent staining of LC3 (red). Data is representative of two independent experiments. **(B)** Electron micrograph of *Aspergillus* cupping. The bottom panel is zoomed in from the area indicated in the top panel. Data is representative of two independent experiments. **(C)** Expression of LC3 and p62 proteins by western blot in whole cell lysates of D1 cells stimulated for 24 h with *A. fumigatus* swollen conidia. Densitometry for LC3 was performed on the LC3-II band to measure LC3-II turnover, while densitometry analysis of p62 was done on the upper band corresponding to its expected molecular weight of 60kD. Data is representative of two biological replicates**. (D)** Multilamellar bodies (encircled in yellow dashed lines) observed by electron microscopy in *A. fumigatus* swollen conidia-stimulated D1 cells and labeled for LAMP-1 or Leucine-rich repeat kinase 2 with 5 nm or 15 nm gold particles (size of particles indicated in superscript). Cells were incubated with fungi for 3 h. Size of scale bars are as indicated. Abbreviations: Untreated (Unt); *A. fumigatus swollen conidia* (A-sw).

Lysosomes are known to be involved in the maturation of autophagic vesicles. Given that autophagy is activated by β-glucan (27) and *A. fumigatus* (28, 29), we investigated autophagy in D1 cells stimulated with *Aspergillus* swollen conidia in order to establish a possible role of the autophagic response in the regulation of the NFAT1 translocation. D1 cells stimulated with swollen conidia showed the formation of LC3-positive phagosomes [LC3 associated phagocytosis (LAPosome)] (Figure 3A) similar to that induced by β-glucan (27) and *A. fumigatus* (30) in other studies, although germinated A-sw conidia were only partially engulfed. Indeeed, by electron microscopy, it was clear that the resulting membrane cupping was not formed by the double-membranes characteristic of classical autophagy (Figure 3B) as previously shown (30). Although images (Figure 3B) show partial fungal internalization, time-lapse imaging of DCs demonstrates the bright field on the EVOS FL Auto Cell Imaging System a complete process of phagocytosis (Supplementary Video S1 in Supplementary Material). Taken together, the observations from immunofluorescent staining of LC3 and electron microscopy indicate that the autophagic response induced by *A. fumigatus* swollen conidia in DCs is non-canonical, as reported for conidia (31). LC3-I to LC3-II conversion and higher LC3-II turnover was indeed observed in *A. fumigatus*-stimulated DCs, and p62 was not degraded (Figure 3C; Figure S1C in Supplementary Material). Our results indicate that swollen conidia induce non-canonical autophagy in DCs. In addition, we found that there was an increase of subcellular multivesicular structures present in DCs stimulated with swollen conidia as revealed by electron microscopy. Multivesicular structures express LAMP-1 as well as LRRK2 (Figure 3D). These structures are lysosomal organelles comprising of multiple concentric layers of membrane, that have been shown to require autophagy for its formation (32). Therefore, taking into account our findings, the germinated form of *Aspergillus* is able to trigger the non-canonical autophagic response together with formation of multivesicular structure formation LRRK2^+^ in DCs.

### Ca^2+^/NFAT/IL-2 Axis Is Affected by Early Autophagic Events, Phagocytosis, and Lysosomal Maturation

**Figure 4:**
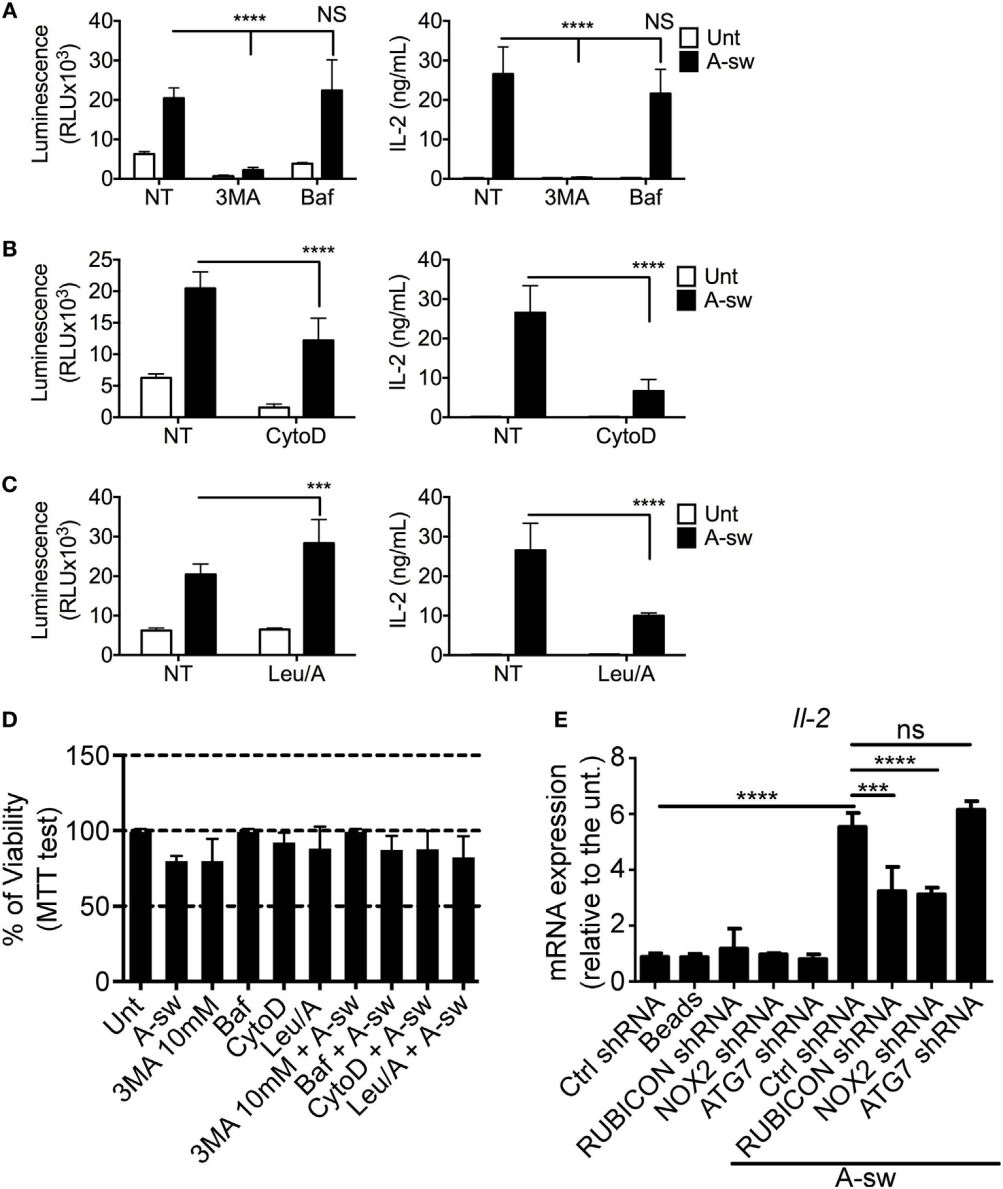
Ca^2+^-NFAT-IL-2 axis is affected by early autophagic events, phagocytosis and LAP. nuclear factor of activated T cells translocation and IL-2 production of NFAT-luciferase reporter D1 cells that have been stimulated with A-sw for 6 h in the presence and absence of drugs inhibiting specific cellular processes. **(A)** Fungus-stimulated D1 cells in the presence and absence of the type III Phosphatidylinositol 3-kinase inhibitor 3MA, 10 mM, or the vacuolar H + ATPase inhibitor, Baf (50 nM), which inhibits the early and late stage of autophagy respectively. **(B)** Fungus-stimulated D1 cells in the presence or absence of the phagocytosis inhibitor, CytoD (2 µg/mL). **(C)** D1 cells stimulated with fungi in the presence or absence of the lysosomal acidification inhibiting combination of Leu/A. Data is displayed as the mean ± SD of five (in the case of Leu/A) or eight biological replicates. **(D)** Viability of D1 cells treated with fungus, drugs, or a combination of the both. **(E)** mRNA expression of IL-2 in D1 cells silenced for various mRNA regulating LAP in the presence or absence of *A. fumigatus* swollen conidia. Data is displayed as means ± SD of three biological replicates. Differences found to be statistically significant by one-way ANOVA with Bonferroni’s Multiple Comparison post-test are indicated (NS, non-significant; ***, *p* < 0.001; ****, *p* < 0.0001). Abbreviations: Untreated (Unt); *A. fumigatus swollen conidia* (A-sw); Vehicle non-treated control (NT); 3-methyladenine (3MA); Bafilomycin (Baf); Cytochalasin D (CytoD), combination of Leupeptin and ammonium chloride (Leu/A).

Based on the previous findings, two types of autophagy inhibitors, 3MA and Baf were used to investigate whether NFAT1 translocation is mediated by the autophagic response to *A. fumigatus*. As the initiation of autophagosome formation requires Class III PI3 kinase activity, 3MA, a PI3 kinase inhibitor, functions by inhibiting autophagy in the early stages of its initiation. Baf inhibits the last step of autophagosome cargo degradation by preventing the autophagosome–lysosome fusion as well as lysosome acidification (33). We found that NFAT1 translocation and IL-2 cytokine production in DCs stimulated with *Aspergillus* germinated conidia were significantly decreased upon inhibition of the early stages of autophagy by 3-MA, but not with an inhibitor of the late stage of autophagy, Baf (Figure 4A). In addition, NFAT pathway in stimulated DCs was also significantly decreased when cells were treated with the phagocytosis inhibitor, CytoD (Figure 4B). DCs were also treated with a combination of Leupeptin and ammonium chloride (Leu/A) to inhibit lysosomal maturation by preventing acidification, and it was observed that NFAT1 translocation increased, while IL-2 production unexpectedly decreased in drug-exposed DCs in response to fungi (Figure 4C). In order to understand whether those variations were due to a reduced cell viability, we performed the MTT proliferation assay on all the conditions tested above (Figure 4D). Pivotally, cells were not affected in terms of viability by the different treatments (Figure 4D).

Finally, since more recently, non-canonical autophagy, also defined as LAP, has been extensively described for infection with *A. fumigatus* (29, 31), we performed shRNA of the main key players (*Rubicon, Nox2, Atg7*) of LAP on D1 cells upon *Aspergillus* treatment and we went to analyze IL-2 transcription as a key response downstream of NFAT translocation (Figure 4E; Figure S2 in Supplementary Material). Results here indicate that shRNA of *Rubicon* and *Nox2* mRNA, lead to a significant decrease of IL-2 transcription upon A-sw stimulation. Together, the results show that early events that occur after fungal stimulation, such as phagocytosis and the early part of the autophagy pathway, are important in the activation of the NFAT pathway, but not late-stage autophagy. Moreover, inhibition of LAP is particularly interfering NFAT translocation in DCs although shRNA of *Rubicon* is not affecting the phagocytosis process (Figure S3 in Supplementary Material). Thus, the induction of non-canonical autophagy as well as the downregulation of LRRK2 expression in *A. fumigatus*-stimulated DCs raises the possibility of autophagy being responsible for the degradation of LRRK2, resulting in the activation of NFAT1. This is further supported by the presence of LRRK2 in lysosomic-endosomic structures (Figure 2C) and multilamellar bodies (Figure 3D).

### LRRK2 Deficiency in DCs Leads to Increased NFAT/IL-2 Activation Axis in Response to *A. fumigatus*

**Figure 5:**
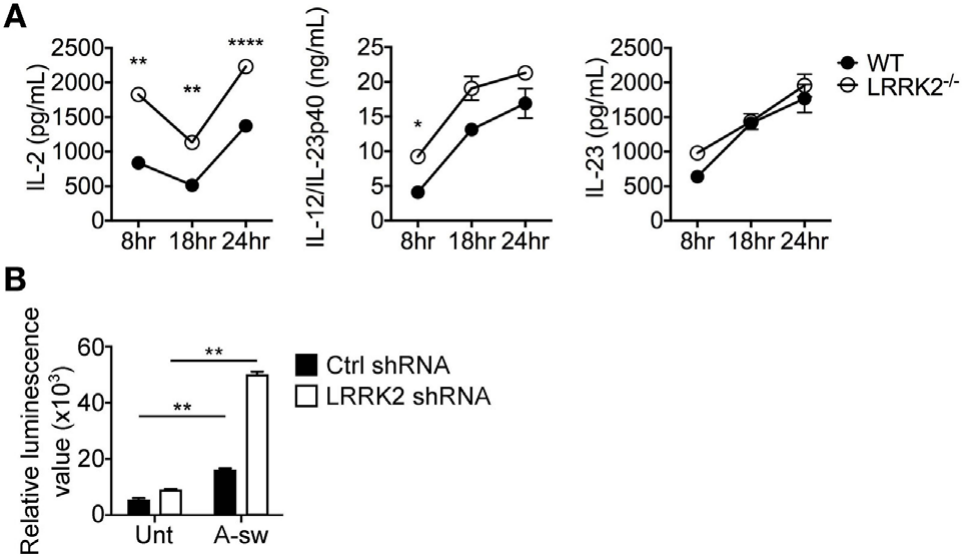
Leucine-rich repeat kinase 2 (LRRK2) deficiency in dendritic cells (DCs) leads to increased IL-2 production and nuclear factor of activated T cells (NFAT) translocation in response to *Aspergillus fumigatus*. **(A)** IL-2, IL-12/IL-23p40, and IL-23 cytokine production of wild-type (closed circles) and LRRK2^−/−^ (open circles) bone marrow-derived DCs in response to fungal stimulation with *A. fumigatus* swollen conidia. Data is displayed as the mean cytokine concentration ± SD of two biological replicates and statistical significance determined by one-way ANOVA with Bonferroni’s Multiple Comparison post-test. **(B)** NFAT translocation measured by luminescence signal in A-sw-stimulated NFAT-luciferase reporter D1 cells upon LRRK2 silencing. Data is displayed as the mean luminescence signal ± SD of three biological replicates and statistical significance determined by Student’s *t*-test. Differences found to be statistically significant are indicated (*, *p* < 0.05; **, *p* < 0.01; ****, *p* < 0.0001). Abbreviations: Untreated (Unt); *A. fumigatus swollen conidia* (A-sw).

Since LRRK2 has been reported as a negative regulator of the NFAT pathway (15), we used LRRK2^−/−^ BMDCs (Figure S4 in Supplementary Material) to measure IL-2 release as well as other two cytokine regulated by NFAT in DCs (34) upon *Aspergillus* germinated spore stimulation. LRRK2^−/−^ BMDCs showed a significant increase in IL-2 production when stimulated with *Aspergillus* swollen conidia at 8, 18, and 24 h, while cytokine as IL-12/IL-23p40 was significantly increased only at 8 h and IL-23 did not show significant changes (Figure 5A). More interestingly, stimulated NFAT-luciferase reporter D1 cells, upon silencing with LRRK2 shRNA and exposed to A-sw showed that NFAT nuclear translocation was also significantly increased (Figure 5B). In these settings, IL-2 was also increased upon A-sw stimulation (data not shown). Therefore, LRRK2 regulates the NFAT pathway in response to the fungus *Aspergillus*. Consequently, LRRK2 deficiency may disentangle a possible dysregulation of the NFAT/IL-2 cascade in response to the germinated fungus *Aspergillus*.

### The Independent Role of LRRK2 in Regulating the NFAT/IL-2 Axis in *Aspergillus*-Stimulated DCs

It has been reported that LRRK2 physically associates with the NRON complex, and that NRON was important for mediating the regulation of NFAT1 nuclear translocation by LRRK2 (15), hence the expression of four members of the NRON complex; NRON, PPP2R1A, CSE1L, and SPAG9 was investigated in DCs in response to *Aspergillus* swollen conidia. Gene expression analysis shows that components of the NRON complex are expressed in DCs, and are negatively regulated by fungal exposure (Figure S5A in Supplementary Material). On the protein level, the expression of the NRON complex components, including PPP2R1A, CSE1L and SPAG9, could also be detected at basal level. Interestingly, *Aspergillus*-stimulated D1 cells showed marked downregulation of the PPP2R1A protein (Figure S5B in Supplementary Material). Altogether, this shows that *Aspergillus* by inducing phagocytosis and LAP may also influence the expression of other components of the NRON complex as for LRRK2 in DCs. In order to investigate whether the NRON complex in DCs was affecting the NFAT/IL-2 axis as shown for LRRK2, D1 cells were knocked down for NRON, IQGAP, CSE1L, and PPP2R1A (a subunit of the PPP2RA protein), or SPAG9 using shRNA-containing lentiviral particles. Silencing efficiency was investigated and the DC clone was selected accordingly (Figure S6 in Supplementary Material).

**Figure 6:**
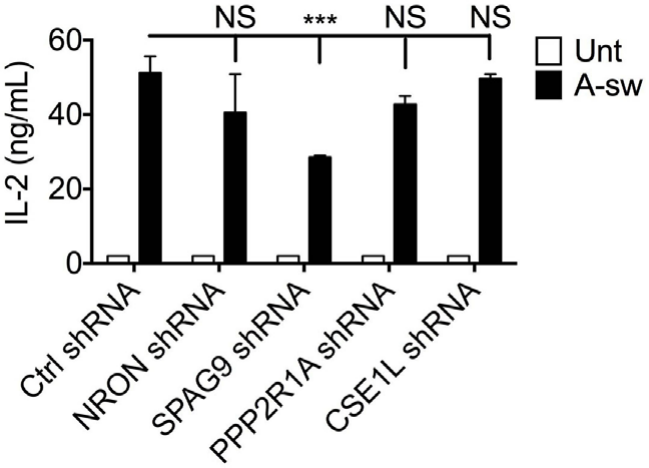
Regulation of IL-2 by NRON complex components. IL-2 cytokine production at 8 h post-exposure from A-sw stimulated D1 cells that were knocked down for NRON, sperm-associated antigen 9, PPP2R1A, or chromosome segregation 1-like through the use of shRNA-lentiviral particles. Data are displayed as the mean ± SD of three replicates. Differences between *Aspergillus*-stimulated cells found to be statistically significant by one-way ANOVA with Bonferroni’s Multiple Comparison post-test are indicated (NS, non-significant; ***, *p* < 0.001). Abbreviations: Untreated (Unt); *A. fumigatus swollen conidia* (A-sw).

Surprisingly, no increase in IL-2 release in response to *Aspergillus* stimulation from silenced D1 cells was observed as we found in LRRK2 deficiency. Interestingly, other cytokines involved in DC response to infections as IL-1β, IL-6, IL12/IL-23p40, IL-22, IL-23, and TNFα are differently affected by silencing the NRON complex components (Figure S7 in Supplementary Material). Regarding IL-2, only knocking down of SPAG9 led to a decrease of IL-2 production by DCs (Figure 6).

**Figure 7:**
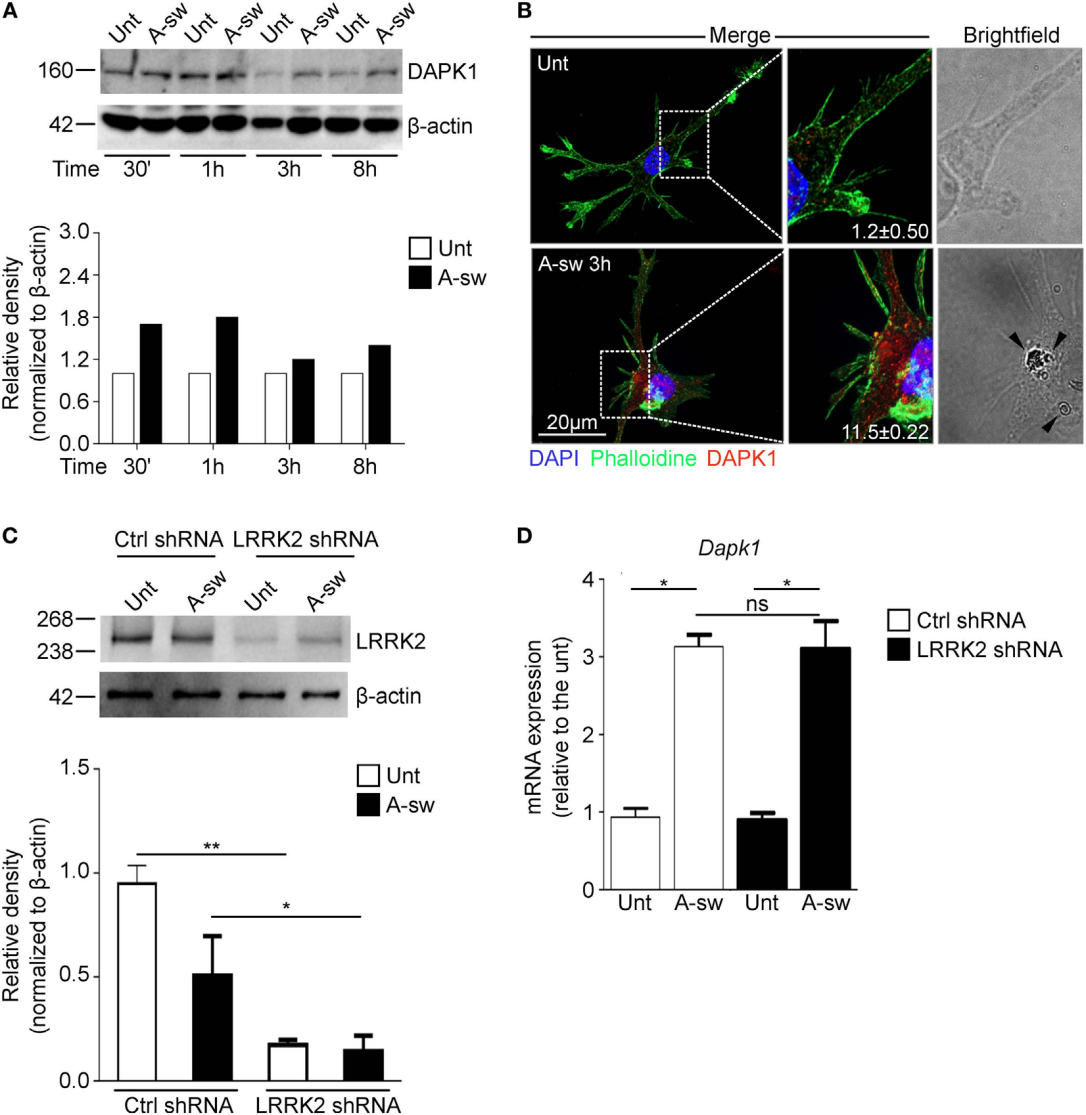
Death-associated protein kinase 1 (DAPK1) expression during LAP is not controlled by Leucine-rich repeat kinase 2 (LRRK2). **(A)** Protein expression of DAPK1 in *A. fumigatus* swollen conidia-stimulated D1 cells. For densitometry analysis, band densities for DAPK1 were normalized to the band density of the housekeeping protein, β-actin, after which the density of the DAPK1 band of the treated sample was compared with that of the untreated sample. Data are representative of three independent experiments. **(B)** Immunofluorescent stanining of DAPK1 and brightfield microscopy of *Aspergillus fumigatus* swollen conidia-stimulated D1 cells. Signal intensity of the DAPK1 was quantified and displayed as mean fluorescence signal ± SD of three biological replicates. Brightfield imaging of the same cell is displayed with arrows indicating the presence of *A. fumigatus* swollen conidia in the cell. **(C)** Knockdown of LRRK2 in D1 cells was assessed by western blot. Data are represented as mean ± SD of three biological replicates and statistical significance determined by two way ANOVA **(D)** mRNA expression of DAPK1 in fungal-treated D1 cells that have been silenced for LRRK2. Data are displayed as the mean mRNA expression ± SD of three biological replicates and statistical significance determined by two way ANOVA. Differences found to be statistically significant are indicated (*, *p* < 0.05; **, *p* < 0.01). Abbreviations: *A. fumigatus swollen conidia* (A-sw); Untreated (Unt); D1 cells transduced with non-targeting control shRNA (Ctrl).

Finally, since recently, a mechanism by which inflammation is regulated during LAP through the death-associated protein kinase 1 (DAPK1) has been described in macrophages (31), we investigated a possible cross-regulation between these two proteins during LAP. Moreover, *Dapk1* and *Lrrk2* are both target genes of IFN-γ in myeloid cells (10, 35). Therefore, we have analyzed DAPK1 expression in DCs (Figures 7A,B), and we found that DAPK1 protein level increases in response to A-sw (Figure 7A) differently from LRRK2 in DCs (Figure 1A). However, DCs treated with LRRK2 shRNA (Figure 7C), do not show any significant differential expression of *Dapk1* (Figure 7D) as well as DAPK1 shRNA treated DCs show similar *Lrrk2* expression than Ctrl shRNA (data not shown). In conclusion, our results underline a more prominent and independent (from NRON components) role of LRRK2 in regulating the NFAT1 translocation to the nucleus and in regulating IL-2 cytokine release in DCs (Figure S8 in Supplementary Material).

## Discussion

Leucine-rich repeat kinase 2 was first discovered to play a role in Parkinson’s disease (2), and it has been shown to be involved in various signaling pathways and cellular processes. In addition, LRRK2 is genetically associated with other inflammatory diseases (15, 36, 37) and modulated in inflammation by PAMPs (10, 15). Also, LRRK2 was reported to be a negative regulator of the NFAT pathway in BMDMs (15), activated downstream of Dectin-1 by fungi (18). Interestingly, LRRK2 has been investigated recently in immune response to pathogens (10, 17). However, as of now the role of LRRK2 protein in fungal immunity has not been explored.

In support of the observations of *Liu* et al. (15), our study here demonstrates that LRRK2 is indeed involved as a negative regulator of the NFAT pathway in DC response to fungi.

At resting state in DCs, we found that LRRK2 localizes to endosomes, accordingly to recent studies, where LRRK2 interaction with the endocytic network have been recently demonstrated (24, 38, 39). Liu et al. (15) reported LRRK2 to be bound to 5 of the 11 proteins of the NRON complex. In view of these reports, the localization of LRRK2 and possibly also other NRON complex components to endosomes at steady state could serve to regulate NFAT, and this is possibly accomplished by sequestering it at the endosomal membrane.

In this study, four components, including NRON, PPP2R1A, CSE1L, and SPAG9 of the NRON complex were also investigated, and it was found that expression levels of NRON and PPP2R1A in particular, were downregulated in DCs in response to *Aspergillus* stimulation as LRRK2. This is in line with what has been reported that the NRON complex, together with LRRK2, mediates NFAT translocation regulation (15) since a downregulation of NRON complex components would likely lead to a dissociation of this regulatory complex. A knockdown of these components was carried out to investigate whether other than LRRK2 other components may affect NFAT nuclear translocation in DCs. IL-2 production from *Aspergillus*-stimulated DCs knocked down for any of these components was found not to be significantly affected, or was decreased as in the case when SPAG9 was knocked down. This indicates that perhaps NRON, PPP2R1A, and CSE1L alone are not sufficient to regulate the NFAT/IL-2 axis. SPAG9, on the other hand, individually could be positively regulating the NFAT/IL-2 axis, rather than inhibiting it.

More interestingly, the individual components of the NRON complex were regulating DC cytokine response differently, implying that they may not work in concert.

For SPAG9, CSE1L, and PPP2R1A, besides being known to be part of the NRON complex (15, 40), their reported functions were not originally concerning the immune system. SPAG9 has been proposed as a biomarker for diagnosis in carcinoma of the breast (41), endometrium (42), cervix (43), thyroid (44), and colon (45), and has been proposed to be involved in the tumorogenesis and growth. CSE1L is a nuclear exportin protein that is involved in the cell cycle, and SPAG9 has also been associated with various carcinomas [as reviewed in Behrens et al. (46)]. PPP2R1A is a subunit of protein phosphatase 2A (PP2A). PP2A has been implicated in meiosis and mitosis in numerous studies (47, 48) and is currently being explored as a treatment target for pancreatic cancer (49). More related to the context of this study, PP2A proteins have been shown to interact with signaling pathways, such as the TLR-TRIF signaling (50), Ras signaling (51), as well as Ca^2+^/Calmodulin-dependent protein kinase B/Akt (52). In relation to neurological disorders, PP2A has been suggested as a possible treatment target for neurological disorders Alzheimer’s disease (53) and has been recently implicated in Tau pathology of Parkinson’s disease (54). This study hints that the function of SPAG9, CSE1L, and PPP2R1A may also be related to the immune response to pathogens.

It has been shown that LRRK2 localizes to lysosomes and can be degraded by CMA in neurons (23). In a similar way, this study here shows that LRRK2 is localized to endosomes and lysosomes in steady state DCs, and that *Aspergillus* is able to induce a non-canonical type of autophagy in DCs that is reminiscent of LC3-positive phagosomes, previously reported (27, 30, 31, 55).

The use of transmission electron microscopy allowed the observation of LRRK2-positive multilamellar body formation in *Aspergillus*-stimulated DCs, further strengthening the connection of LRRK2 with lysosomes and autophagy. Multilamellar bodies are reported to be part of the lysosomal pathway and its formation is dependent on autophagy and lysosomal degradation (32), and the localization of LRRK2 to these structures is supported by what has been previously reported in cultured human cells (25).

Interestingly, GSK3-β, a protein involved in phosphorylating NFAT, has also been shown to localize to the endosomal membrane and this is thought to serve to isolate it from interaction with other signaling components (26).

How the NFAT pathway is influenced by the events following the engagement of Dectin-1 by *Aspergillus* in DCs was also investigated here. The dependency of phagocytosis on IL-2 production in response to particulate β-glucan in DCs has been previously demonstrated (19), hence in this study the finding that the NFAT/IL-2 axis in response to *Aspergillus* is also dependent on this cellular process was expected. What was interesting was that early, but not late autophagic events are also required to activate the NFAT/IL-2 pathway.

Autophagy also occurs in macrophages, and with this respect Ma et al. (27) and Kyrmizi et al. (30) have both demonstrated that the formation of LC3-positive phagosomes also occur in macrophages incubated with fungi, and that the recruitment of LC3 is important for macrophage signaling and function in response to fungi. In particular, Kyrmizi, Gresnigt (30) show that the recruitment of LC3 is needed for macrophage ROS production and killing of *Aspergillus* spores.

In this study, we have also evaluated whether DAPK1 may contribute to regulating LRRK2 function (and vice versa), since both are regulated during LAP and concur to the regulation of the inflammatory response during fungal LAP. To this purpose, we have resorted to shRNA of LRRK2 and analyzed DAPK1 expression.

To conclude, our study has shown that DCs express LRRK2 and that it negatively regulates the NFAT pathway activated in response to *Aspergillus*. Upon *Aspergillus* binding to Dectin-1 in DCs, non-canonical autophagy, as well as multilamellar body formation is triggered. Taking into account the findings of previously published reports, the sequestration of LRRK2 in the multilamellar bodies could lead to the dissociation of the NRON complex. Therefore, the role of the NRON complex in immune response of DCs to *Aspergillus* has been shown to be more complex than previously thought, and their interaction with other signaling pathways activated in the immune response will add a new dimension to their currently known cellular functions. Given that DCs have an important role in the immune system as antigen presenting cells and initiating the appropriate adaptive immune response, future *in vivo* studies could elucidate better the immunological function of LRRK2 and the NRON complex eventually in T cell priming. The knowledge obtained from such studies not only sheds light on the control of the NFAT pathway, but also could have implications in other diseases associated with LRRK2, such as Crohn’s disease, IBD, and Parkinson’s disease.

## Ethics Statement

All experiments and procedures were approved by the IACUC of A*STAR (Biopolis, Singapore) (Authorization No.: IACUC 110626) in accordance with the guidelines of the AVA and the NACLAR of Singapore.

## Author Contributions

AW designed the study, conducted, analyzed experiments, and wrote the manuscript. VO performed western blotting and immunofluorescence experiments. GP performed qPCR data. AL performed ELISA; MP analyzed western blotting data. JF performed the LRRK2^−/−^ bone marrow expansion experiments. HT generated the D1 NFAT translocation-firefly luciferase reporter cells. PR-C supervised the project. TZ conceived, coordinated the project, and wrote the manuscript.

## Conflict of Interest Statement

The authors declare that the research was conducted in the absence of any commercial or financial relationships that could be construed as a potential conflict of interest.

## Supplementary Material

Video S1 BMDCs phagocytosis of Aspergillus swollen conidia. Representative example of time-lapse imaging data of in vitro mouse BMDCs. BMDCs were infected with Aspergillus swollen conidia and imaged over a period of 1 hour to identify the process of phagocytosis. Images were captured every 2 minutes in the bright field channel on the EVOS FL Auto Cell Imaging System. A movie was then generated from the images at rate of 4 frame/sec.

## References

[1] Paisán-Ruíz C Jain S Evans E Gilks W Simón J van der Brug M Cloning of the gene containing mutations that cause PARK8-linked Parkinson’s disease. Neuron (2004) 44(4):595–600. 10.1016/j.neuron.2004.10.023 15541308

[2] Funayama M Hasegawa K Kowa H Saito M Tsuji S Obata F. A new locus for Parkinson’s disease (PARK8) maps to chromosome 12p11.2-q13.1. Ann Neurol (2002) 51(3):296–301. 10.1002/ana.10113 11891824

[3] Mata I Wedemeyer W Farrer M Taylor J Gallo K. LRRK2 in Parkinson’s disease: protein domains and functional insights. Trends Neurosci (2006) 29(5):286–93. 10.1016/j.tins.2006.03.006 16616379

[4] Jorgensen N Peng Y Ho CC-Y Rideout H Petrey D Liu P The WD40 domain is required for LRRK2 neurotoxicity. PLoS One (2009) 4(12):e8463. 10.1371/journal.pone.0008463 20041156 PMC2794542

[5] Smith W Pei Z Jiang H Moore D Liang Y West A Leucine-rich repeat kinase 2 (LRRK2) interacts with parkin, and mutant LRRK2 induces neuronal degeneration. Proc Natl Acad Sci U S A (2005) 102(51):18676–81. 10.1073/pnas.0508052102 16352719 PMC1317945

[6] West A Moore D Choi C Andrabi S Li X Dikeman D Parkinson’s disease-associated mutations in LRRK2 link enhanced GTP-binding and kinase activities to neuronal toxicity. Hum Mol Genet (2007) 16(2):223–32. 10.1093/hmg/ddl471 17200152

[7] Liu B. Modulation of microglial pro-inflammatory and neurotoxic activity for the treatment of Parkinson’s disease. AAPS J (2006) 8(3):21. 10.1208/aapsj080369 17025278 PMC2668934

[8] Whitton P. Inflammation as a causative factor in the aetiology of Parkinson’s disease. Br J Pharmacol (2007) 150(8):963–76. 10.1038/sj.bjp.0707167 17339843 PMC2013918

[9] Gerlach OH Winogrodzka A Weber W. Clinical problems in the hospitalized Parkinson’s disease patient: systematic review. Mov Disord (2011) 26(2):197–208. 10.1002/mds.23449 21284037 PMC3130138

[10] Gardet AS Benita Y Li C Sands B Ballester I Stevens C LRRK2 is involved in the IFN-gamma response and host response to pathogens. J Immunol (2010) 185(9):5577–85. 10.4049/jimmunol.1000548 20921534 PMC3156100

[11] Hakimi M Selvanantham T Swinton E Padmore R Tong Y Kabbach G Parkinson’s disease-linked LRRK2 is expressed in circulating and tissue immune cells and upregulated following recognition of microbial structures. J Neural Transm (Vienna) (2011) 118(5):795–808. 10.1007/s00702-011-0653-2 21552986 PMC3376651

[12] Moehle M Webber P Tse T Sukar N Standaert D DeSilva T LRRK2 inhibition attenuates microglial inflammatory responses. J Neurosci (2012) 32(5):1602–11. 10.1523/JNEUROSCI.5601-11.2012 22302802 PMC3532034

[13] Gillardon F Schmid R Draheim H. Parkinson’s disease-linked leucine-rich repeat kinase 2(R1441G) mutation increases proinflammatory cytokine release from activated primary microglial cells and resultant neurotoxicity. Neuroscience (2012) 208:41–8. 10.1016/j.neuroscience.2012.02.001 22342962

[14] Kim B Yang MS Choi D Kim JH Kim HS Seol W Impaired inflammatory responses in murine Lrrk2-knockdown brain microglia. PLoS One (2012) 7(4):e34693. 10.1371/journal.pone.0034693 22496842 PMC3322140

[15] Liu Z Lee J Krummey S Lu W Cai H Lenardo M. The kinase LRRK2 is a regulator of the transcription factor NFAT that modulates the severity of inflammatory bowel disease. Nat Immunol (2011) 12(11):1063–70. 10.1038/ni.2113 21983832 PMC4140245

[16] Macian F. NFAT proteins: key regulators of T-cell development and function. Nat Rev Immunol (2005) 5(6):472–84. 10.1038/nri1632 15928679

[17] Liu W Liu X Li Y Zhao J Liu Z Hu Z LRRK2 promotes the activation of NLRC4 inflammasome during *Salmonella typhimurium* infection. J Exp Med (2017) 214(10):3051–66. 10.1084/jem.20170014 28821568 PMC5626397

[18] Goodridge HS Simmons RM Underhill DM Dectin-1 stimulation by *Candida albicans* yeast or zymosan triggers NFAT activation in macrophages and dendritic cells. J Immunol (2007) 178(5):3107–15. 10.4049/jimmunol.178.5.3107 17312158

[19] Fric J Zelante T Ricciardi-Castagnoli P Phagocytosis of particulate antigens – all roads lead to calcineurin/NFAT signaling pathway. Front Immunol (2014) 4:513 10.3389/fimmu.2013.00513 24409187 PMC3885923

[20] Zelante T Wong AY Ping TJ Chen J Sumatoh HR Viganò E CD103(+) dendritic cells control Th17 cell function in the lung. Cell Rep (2015) 12(11):1789–801. 10.1016/j.celrep.2015.08.030 26365185

[21] Winzler C Rovere P Rescigno M Granucci F Penna G Adorini L Maturation stages of mouse dendritic cells in growth factor-dependent long-term cultures. J Exp Med (1997) 185(2):317–28. 10.1084/jem.185.2.317 9016880 PMC2196118

[22] Graham JM Isolation of lysosomes from tissues and cells by differential and density gradient centrifugation. Curr Protoc Cell Biol (2001) Chapter 3:Unit 3.6. 10.1002/0471143030.cb0306s07 18228358

[23] Orenstein S Kuo SH Tasset I Arias E Koga H Fernandez-Carasa I Interplay of LRRK2 with chaperone-mediated autophagy. Nat Neurosci (2013) 16(4):394–406. 10.1038/nn.3350 23455607 PMC3609872

[24] Schreij AM Chaineau M Ruan W Lin S Barker PA Fon EA LRRK2 localizes to endosomes and interacts with clathrin-light chains to limit Rac1 activation. EMBO Rep (2015) 16(1):79–86. 10.15252/embr.201438714 25427558 PMC4304731

[25] Alegre-Abarrategui J Christian H Lufino M Mutihac R Venda L Ansorge O LRRK2 regulates autophagic activity and localizes to specific membrane microdomains in a novel human genomic reporter cellular model. Hum Mol Genet (2009) 18(21):4022–34. 10.1093/hmg/ddp346 19640926 PMC2758136

[26] Pálfy M Reményi A Korcsmáros T. Endosomal crosstalk: meeting points for signaling pathways. Trends Cell Biol (2012) 22(9):447–56. 10.1016/j.tcb.2012.06.004 22796207 PMC3430897

[27] Ma J Becker C Lowell C Underhill D. Dectin-1-triggered recruitment of light chain 3 protein to phagosomes facilitates major histocompatibility complex class II presentation of fungal-derived antigens. J Biol Chem (2012) 287(41):34149–56. 10.1074/jbc.M112.382812 22902620 PMC3464523

[28] De Luca A Iannitti R Bozza S Beau R Casagrande A D’Angelo C CD4(+) T cell vaccination overcomes defective cross-presentation of fungal antigens in a mouse model of chronic granulomatous disease. J Clin Invest (2012) 122(5):1816–31. 10.1172/JCI60862 22523066 PMC3336987

[29] Martinez J Malireddi RK Lu Q Cunha LD Pelletier S Gingras S Molecular characterization of LC3-associated phagocytosis reveals distinct roles for Rubicon, NOX2 and autophagy proteins. Nat Cell Biol (2015) 17(7):893–906. 10.1038/ncb3192 26098576 PMC4612372

[30] Kyrmizi I Gresnigt MS Akoumianaki T Samonis G Sidiropoulos P Boumpas D Corticosteroids block autophagy protein recruitment in *Aspergillus fumigatus* phagosomes via targeting dectin-1/Syk kinase signaling. J Immunol (2013) 191(3):1287–99. 10.4049/jimmunol.1300132 23817424 PMC3883106

[31] Oikonomou V Moretti S Renga G Galosi C Borghi M Pariano M Noncanonical fungal autophagy inhibits inflammation in response to IFN-gamma via DAPK1. Cell Host Microbe (2016) 20(6):744–57. 10.1016/j.chom.2016.10.012 27889463 PMC5161749

[32] Hariri M Millane G Guimond MP Guay G Dennis JW Nabi IR. Biogenesis of multilamellar bodies via autophagy. Mol Biol Cell (2000) 11(1):255–68. 10.1091/mbc.11.1.255 10637306 PMC14772

[33] Mizushima N Yoshimori T Levine B. Methods in mammalian autophagy research. Cell (2010) 140(3):313–26. 10.1016/j.cell.2010.01.028 20144757 PMC2852113

[34] Yu HB Yurieva M Balachander A Foo I Leong X Zelante T NFATc2 mediates epigenetic modification of dendritic cell cytokine and chemokine responses to dectin-1 stimulation. Nucleic Acids Res (2015) 43(2):836–47. 10.1093/nar/gku1369 25550437 PMC4333412

[35] Gade P Manjegowda SB Nallar SC Maachani UB Cross AS Kalvakolanu DV. Regulation of the death-associated protein kinase 1 expression and autophagy via ATF6 requires apoptosis signal-regulating kinase 1. Mol Cell Biol (2014) 34(21):4033–48. 10.1128/MCB.00397-14 25135476 PMC4386459

[36] Barrett J Hansoul S Nicolae D Cho J Duerr R Rioux J Genome-wide association defines more than 30 distinct susceptibility loci for Crohn’s disease. Nat Genet (2008) 40(8):955–62. 10.1038/ng.175 18587394 PMC2574810

[37] Törkvist L Halfvarson J Ong R Lördal M Sjöqvist U Bresso F Analysis of 39 Crohn’s disease risk loci in Swedish inflammatory bowel disease patients. Inflamm Bowel Dis (2010) 16(6):907–9. 10.1002/ibd.21105 19760754

[38] Yun HJ Kim H Ga I Oh H Ho DH Kim J An early endosome regulator, Rab5b, is an LRRK2 kinase substrate. J Biochem (2015) 157(6):485–95. 10.1093/jb/mvv005 25605758

[39] Gómez-Suaga P Rivero-Ríos P Fdez E Blanca Ramírez M Ferrer I Aiastui A LRRK2 delays degradative receptor trafficking by impeding late endosomal budding through decreasing Rab7 activity. Hum Mol Genet (2014) 23(25):6779–96. 10.1093/hmg/ddu395 25080504

[40] Willingham A Orth A Batalov S Peters E Wen B Aza-Blanc P A strategy for probing the function of noncoding RNAs finds a repressor of NFAT. Science (2005) 309(5740):1570–3. 10.1126/science.1115901 16141075

[41] Kanojia D Garg M Gupta S Gupta A Suri A Sperm-associated antigen 9, a novel biomarker for early detection of breast cancer. Cancer Epidemiol Biomarkers Prev (2009) 18(2):630–9. 10.1158/1055-9965.EPI-08-0629 19190149

[42] Yu P Yan L Zhang H Lin X Zhao X. Expression and clinical significance of sperm-associated antigen 9 in patients with endometrial carcinoma. Int J Gynecol Cancer (2012) 22(1):87–93. 10.1097/IGC.0b013e3182370f2e 22146769

[43] Garg M Kanojia D Salhan S Suri S Gupta A Lohiya NK Sperm-associated antigen 9 is a biomarker for early cervical carcinoma. Cancer (2009) 115(12):2671–83. 10.1002/cncr.24293 19326449

[44] Garg M Kanojia D Suri S Gupta S Gupta A Suri A. Sperm-associated antigen 9: a novel diagnostic marker for thyroid cancer. J Clin Endocrinol Metab (2009) 94(11):4613–8. 10.1210/jc.2009-0703 19820019

[45] Kanojia D Garg M Gupta S Gupta A Suri A. Sperm-associated antigen 9 is a novel biomarker for colorectal cancer and is involved in tumor growth and tumorigenicity. Am J Pathol (2011) 178(3):1009–20. 10.1016/j.ajpath.2010.11.047 21356354 PMC3069833

[46] Behrens P Brinkmann U Wellmann A. CSE1L/CAS: its role in proliferation and apoptosis. Apoptosis (2003) 8(1):39–44. 10.1023/A:1021644918117 12510150

[47] Hu WW Wang ZB Jiang ZZ Qi ST Huang L Liang QX Scaffold subunit Aalpha of PP2A is essential for female meiosis and fertility in mice. Biol Reprod (2014) 91(1):19. 10.1095/biolreprod.114.120220 24899574

[48] Porter IM Schleicher K Porter M Swedlow JR. Bod1 regulates protein phosphatase 2A at mitotic kinetochores. Nat Commun (2013) 4:2677. 10.1038/ncomms3677 24157919 PMC3826647

[49] Chien W Sun QY Lee KL Ding LW Wuensche P Torres-Fernandez LA Activation of protein phosphatase 2A tumor suppressor as potential treatment of pancreatic cancer. Mol Oncol (2015) 9(4):889–905. 10.1016/j.molonc.2015.01.002 25637283 PMC4387089

[50] Woo CW Kutzler L Kimball SR Tabas I. Toll-like receptor activation suppresses ER stress factor CHOP and translation inhibition through activation of eIF2B. Nat Cell Biol (2012) 14(2):192–200. 10.1038/ncb2408 22231169 PMC3271190

[51] Ory S Zhou M Conrads TP Veenstra TD Morrison DK. Protein phosphatase 2A positively regulates Ras signaling by dephosphorylating KSR1 and Raf-1 on critical 14-3-3 binding sites. Curr Biol (2003) 13(16):1356–64. 10.1016/S0960-9822(03)00535-9 12932319

[52] Fedida-Metula S Feldman B Koshelev V Levin-Gromiko U Voronov E Fishman D. Lipid rafts couple store-operated Ca2+ entry to constitutive activation of PKB/Akt in a Ca2+/calmodulin-, Src- and PP2A-mediated pathway and promote melanoma tumor growth. Carcinogenesis (2012) 33(4):740–50. 10.1093/carcin/bgs021 22287561

[53] Sontag J-MM Sontag E. Protein phosphatase 2A dysfunction in Alzheimer’s disease. Front Mol Neurosci (2014) 7:16. 10.3389/fnmol.2014.00016 24653673 PMC3949405

[54] Arif M Kazim SF Grundke-Iqbal I Garruto RM Iqbal K. Tau pathology involves protein phosphatase 2A in parkinsonism-dementia of Guam. Proc Natl Acad Sci U S A (2014) 111(3):1144–9. 10.1073/pnas.1322614111 24395787 PMC3903234

[55] Nicola AM Albuquerque P Martinez LR Dal-Rosso RA Saylor C Jesus M.De Macrophage autophagy in immunity to *Cryptococcus neoformans* and *Candida albicans*. Infect Immun (2012) 80(9):3065–76. 10.1128/IAI.00358-12 22710871 PMC3418760

