# Supplementary material for: Leucine-Rich Repeat Kinase 2 Controls the Ca2+/Nuclear Factor of Activated T Cells/IL-2 Pathway during Aspergillus Non-Canonical Autophagy in Dendritic Cells
Source: Front Immunol. 2018 Feb 8;9:210. doi: 10.3389/fimmu.2018.00210 (PMC5809498; doi:10.3389/fimmu.2018.00210)
Supplement: Supplementary file 1 [file data_sheet_1.docx]

Supplementary Material

**LRRK2 controls the Ca^2+^/NFAT/IL-2 pathway during**

***Aspergillus* non-canonical autophagy in DCs**

Alicia Yoke Wei Wong,^1,2^ Vasilis Oikonomou,^3^ Giuseppe Paolicelli,^3^ Antonella De Luca,^3^ Marilena Pariano,^3^ Jan Fric,^1,4^ Hock Soon Tay,^1^ Paola Ricciardi-Castagnoli,^1,5^ and Teresa Zelante^1,3*^

^1^Singapore Immunology Network, Agency for Science, Technology and Research, Singapore

^2^National University of Singapore Graduate School for Integrative Sciences and Engineering, National University of Singapore, Singapore

^3^Department of Experimental Medicine, University of Perugia, Perugia, Italy

^4^Center for Translational Medicine (CTM), International Clinical Research Center (ICRC), St. Anne’s University Hospital Brno, Brno, Czech Republic

^5^Toscana Life Sciences Foundation, Siena, Italy

Correspondence:

*Teresa Zelante
University of Perugia
Department of Experimental Medicine
Building C - Level 4
Polo Unico Sant'Andrea delle Fratte
Piazzale Gambuli
06132 Perugia, Italy
Tel. +39.075.585.8238
teresa.zelante@unipg.it


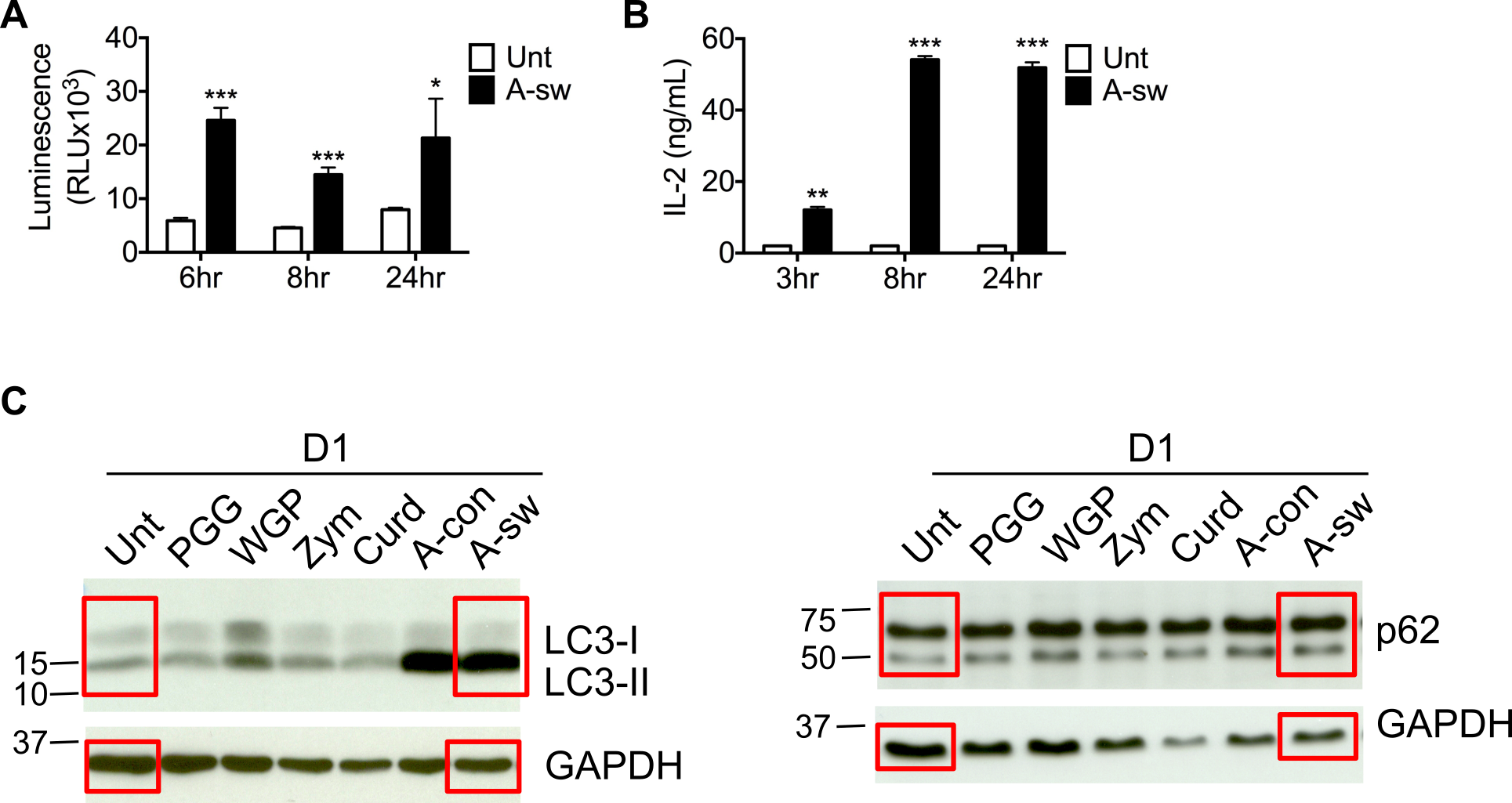


Figure S1: *Aspergillus fumigatus* activation of the Ca^2+^-NFAT-IL-2 response in DCs and LAP. (A) NFAT translocation measured by luminescence signal in fungal-stimulated NFAT-luciferase reporter D1 cells. Data is displayed as the mean luminescence signal±SD of three biological replicates and statistical significance determined by two way ANOVA. (B) IL-2 production from D1 cells stimulated with *A. fumigatus* swollen conidia. Data is displayed as the mean cytokine concentration±SD of two biological replicates. Statistical significance was determined by two way ANOVA. Data is displayed as the mean cytokine concentration±SD of two replicates. (C) Expression of LC3 and p62 proteins by western blot in whole cell lysates of D1 cells stimulated for 24 hours with various stimuli. Data is representative of two biological replicates. Abbreviations used: *A. fumigatus* conidia (A-con); *A. fumigatus* swollen conidia (A-sw); Curdlan (Curd); soluble β-(1, 3)-glucan (PGG); Untreated (Unt); yeast whole glucan particles (WGP); Zymosan (Zym).

**
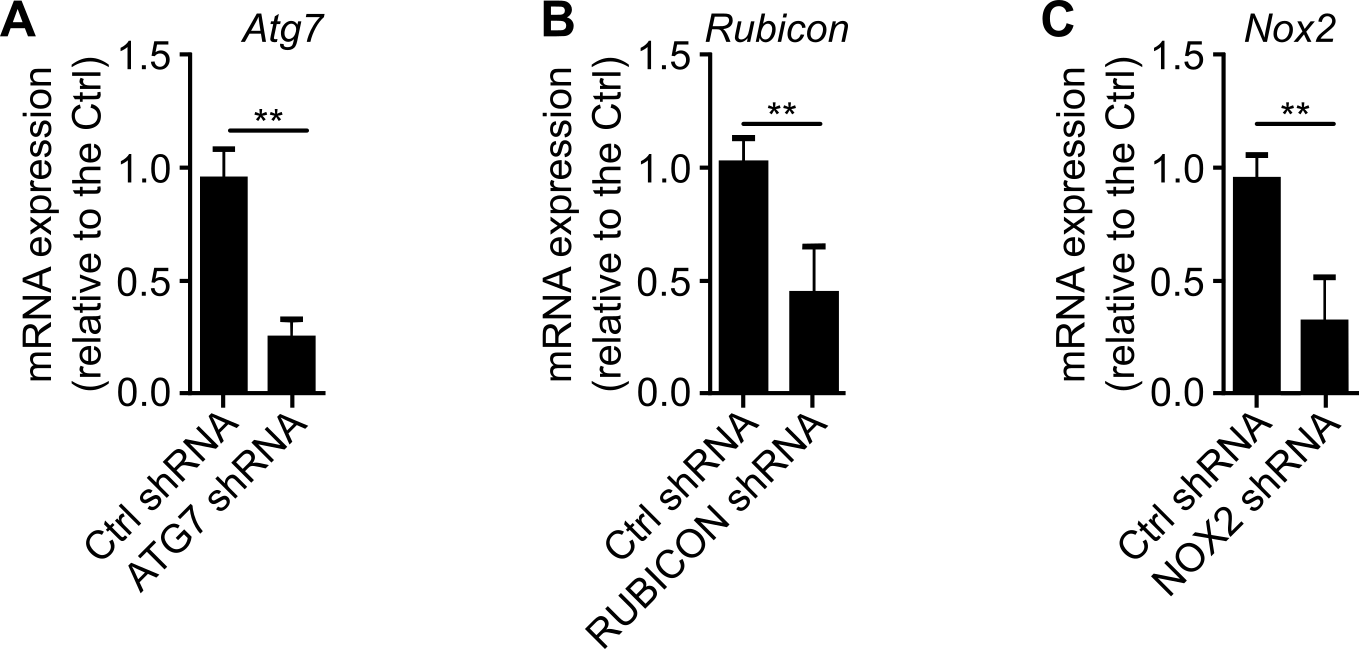
**

Figure S2: Silencing of ATG7, RUBICON, and NOX2 in DCs. (A-C) mRNA expression of LC3-associated phagocytosis proteins in D1 cells silenced for ATG7 (A), RUBICON (B), and NOX2 (C). Data is displayed as the mean mRNA expression±SD of three biological replicates and statistical significance was determined by one-way ANOVA with Bonferroni’s post-test. Differences found to be statistically significant are indicated (* - p<0.05; ** - p<0.01; *** - p<0.001). Abbreviations used: non-targeting control shRNA (Ctrl).

**
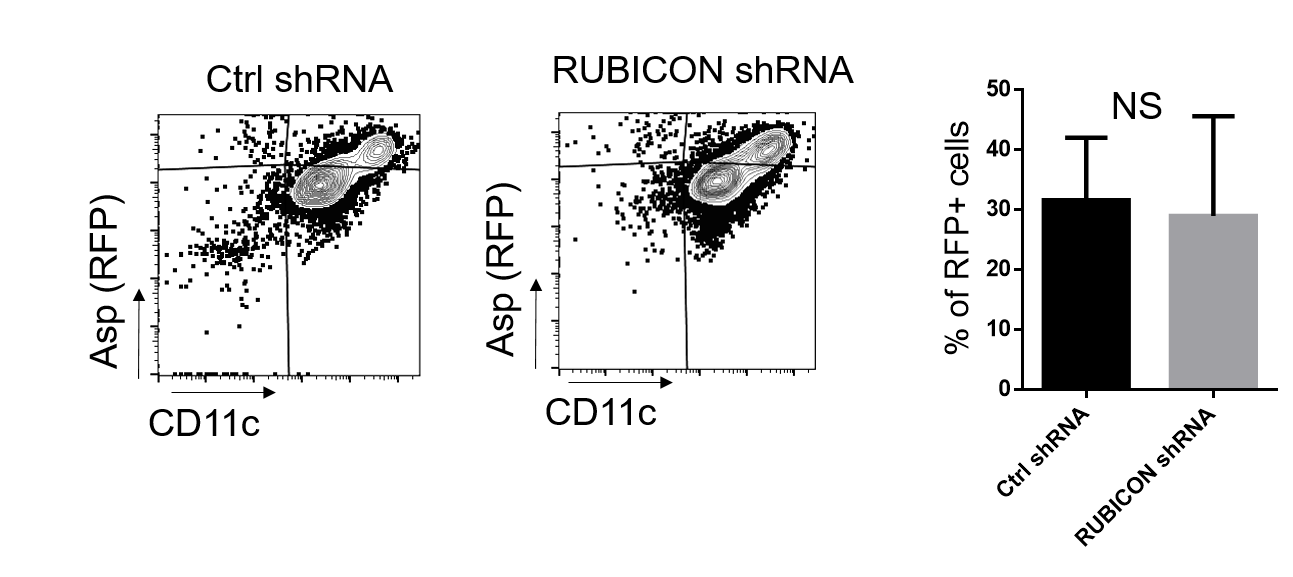
**

Figure S3: Phagocytosis of *Aspergillus* is Rubicon-independent. At 16h post-silencing, BMDCs were treated with *Aspergillus* RFP swollen conidia (ratio 1:1) for 1h analysed by flow cytometry. Data (mean ± s.d.) represent three independent experiments in which technical triplicates of 100,000 cells per sample were acquired using a Fortessa cytometer (BD). Phagocytosis was calculated using flow cytometry analysis (described above). The percentage of phagocytosis equals the number of BMDCs that have engulfed RFP *A. fumigatus*. Abbreviations used: non-targeting control shRNA (Ctrl).


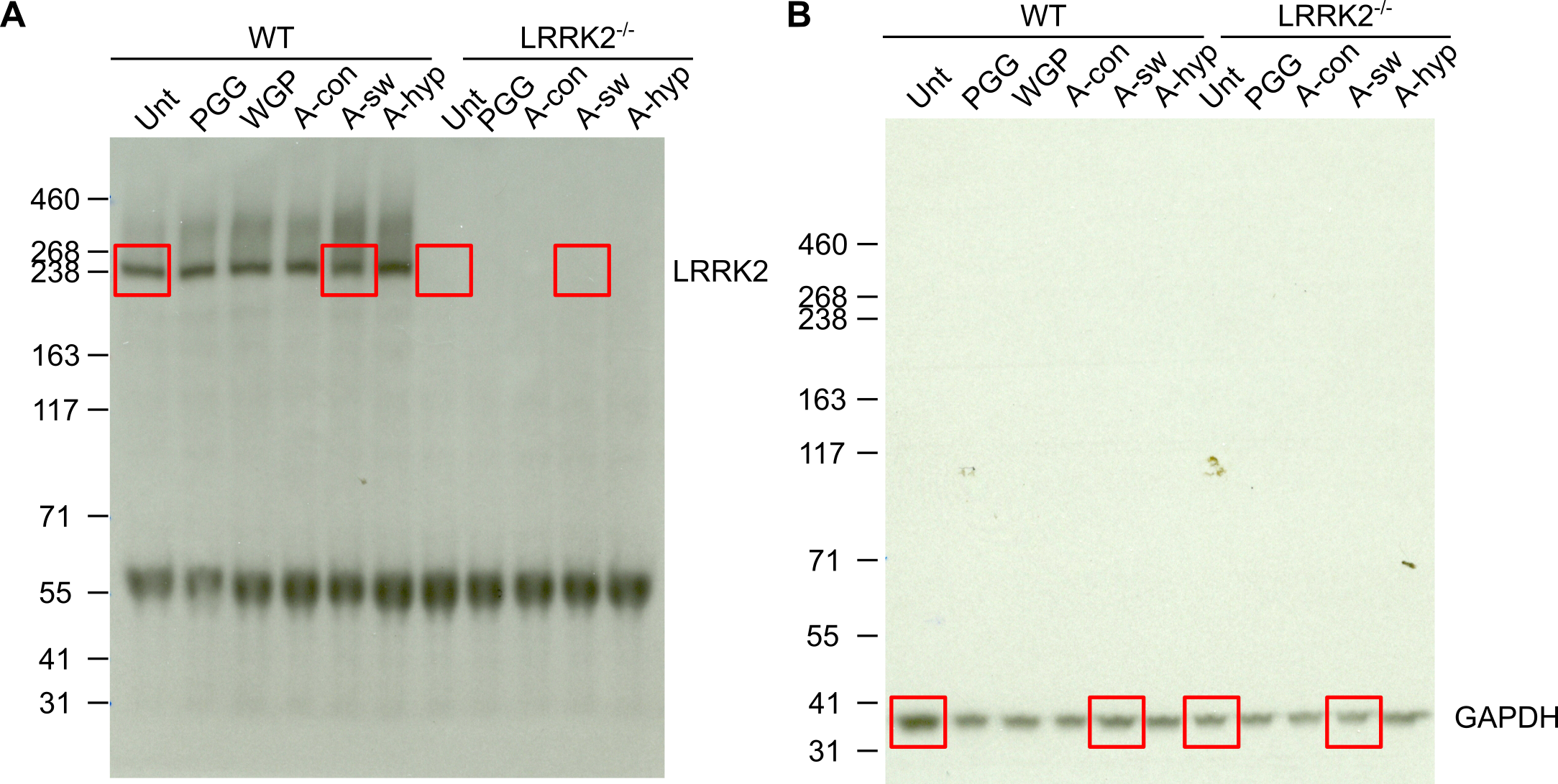


**Figure S4: LRRK2 deficient BMDCs.** Western blot of LRRK2 of wild type and LRRK2^-/-^ BMDCs in response to 3 hours of fungal stimulation with A-sw. Data is representative of two independent experiments. Abbreviations used: *A. fumigatus* conidia (A-con); *A. fumigatus* swollen conidia (A-sw); *A. fumigatus* hyphae (A-hyp); soluble β-(1, 3)-glucan (PGG); Untreated (Unt); yeast whole glucan particles (WGP).


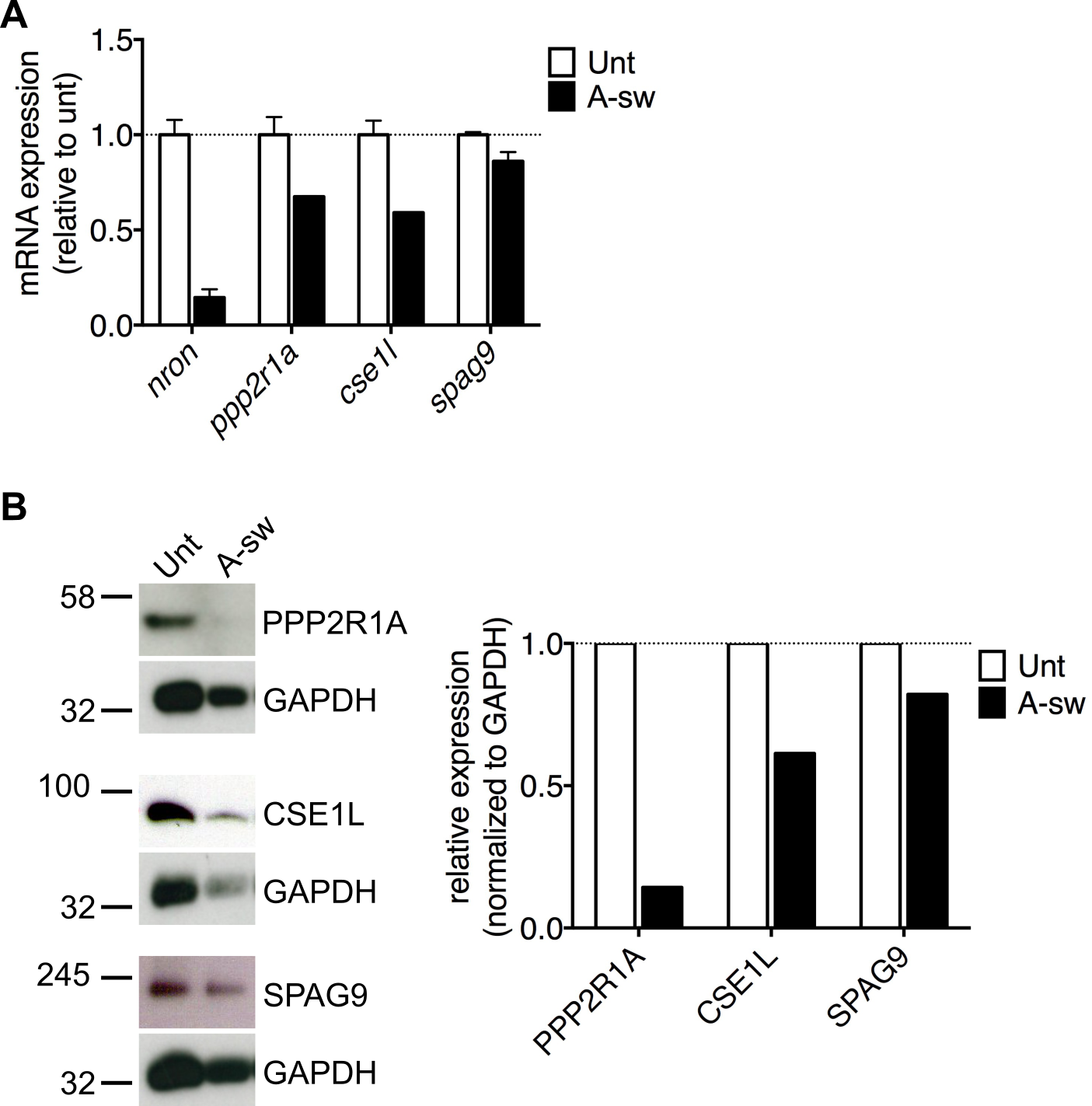


**Figure S5: Expression of NRON complex in DCs.** (A) Gene expression of *nron*, *ppp2r1a*, *cse1l* and *spag9* in D1 cells in response to 8 hour stimulation with *A. fumigatus* swollen conidia. (B) Protein expression of PPP2R1A, CSE1L and SPAG9 by western blot in fungal-stimulated D1 cells for 8 hours. Data are displayed as representative of three biological replicates. Abbreviations used: *A. fumigatus* swollen conidia (A-sw**);** Untreated (Unt).


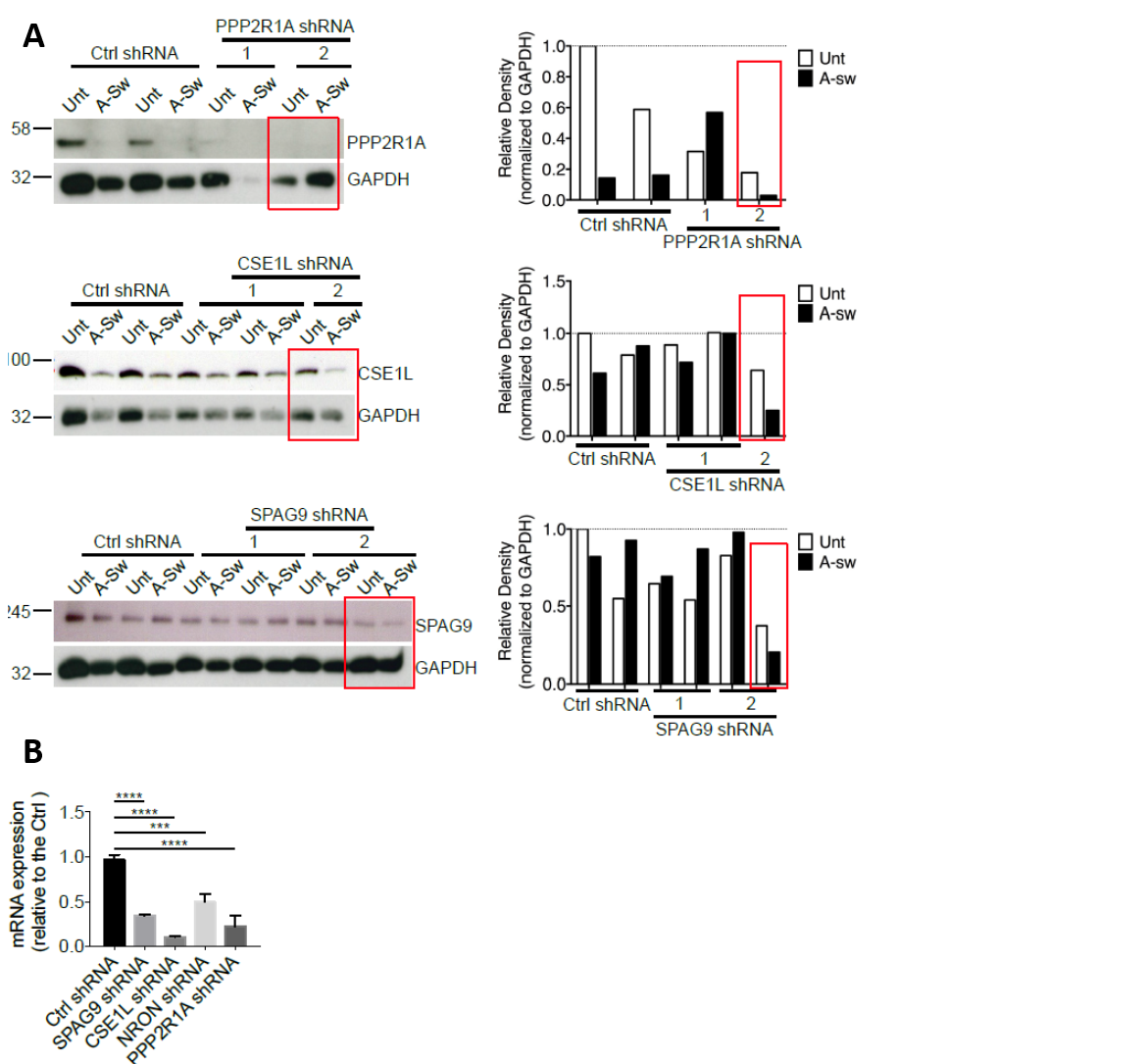


Figure S6: Knock down of components of the NRON complex in DCs. Knock down of *nron*, *ppp2r1a*, *cse1l* and *spag9* in D1 cells were assessed by qPCR and western blot post-selection with 0.5μg/mL of puromycin. (A) Protein expression of PPP2R1A, CSE1L and SPAG9 in A-sw conidia-treated D1 cells tranduced with lentiviruses particles containing shRNA targeting the gene indicated. Replicates with the most degree of knock down were selected for further analysis (highlighted in red). Arabic numerals refer to the two different shRNA sequences used for the silencing. (C) Selected samples from A, were evaluated for knockdown of *spag9*, *cse1l*, *nron*, and *ppp2r1a* by qPCR in unstimulated D1 cells. Data is expressed as the mean relative expression±SD of three replicates and normalized to the GAPDH gene expression level. Abbreviations used: *A. fumigatus* swollen conidia (A-sw); non-targeting control shRNA (Ctrl).


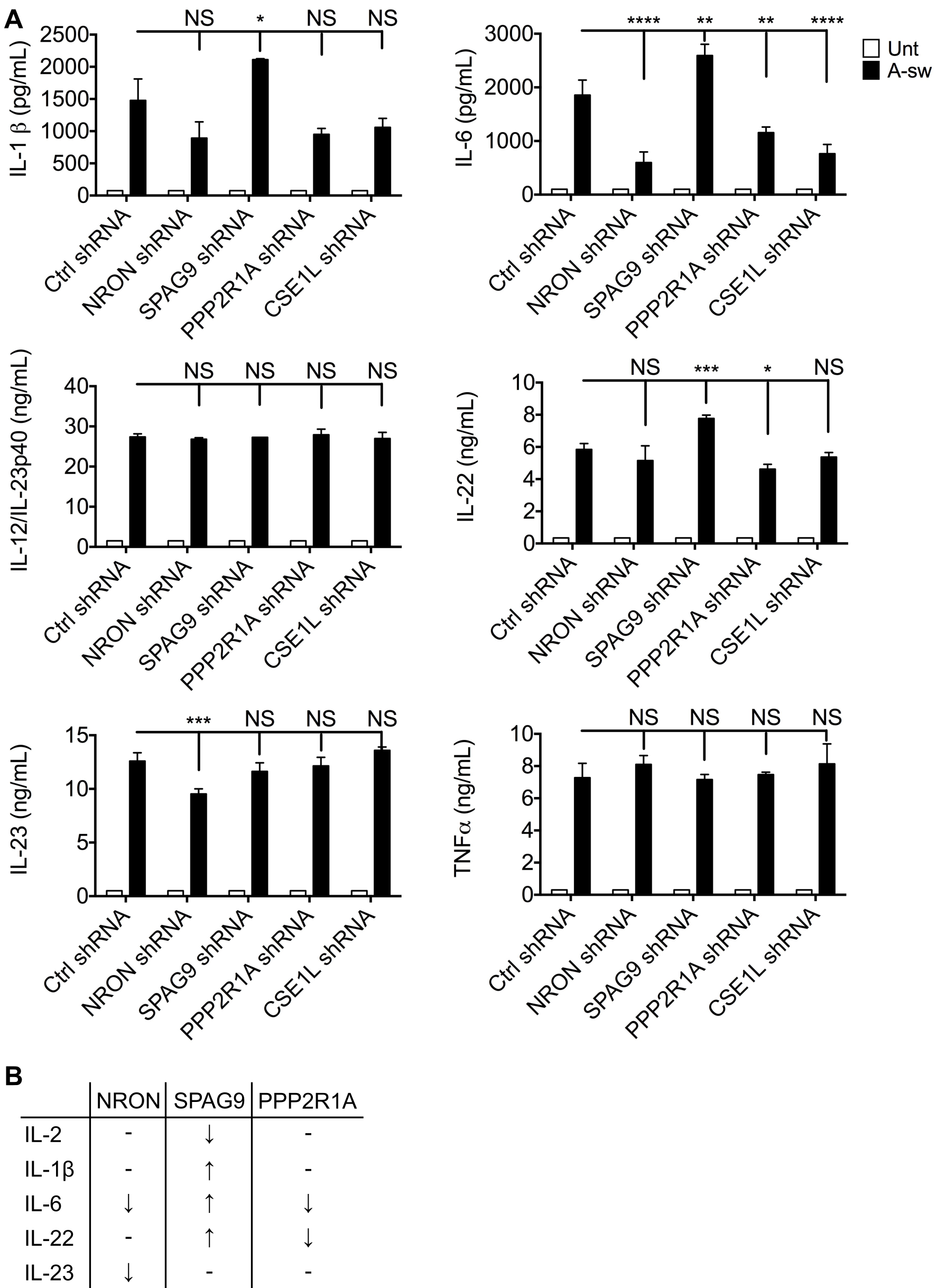


Figure S7: Individual NRON complex components regulate the cytokine response in *Aspergillus*-treated DCs. (A) Cytokine production at 8 hours post-exposure from *A. fumigatus* swollen conidia-stimulated D1 cells that were knocked down for NRON, SPAG9, PPP2R1A or CSE1L through the use of shRNA-lentiviral particles. Data are displayed as the mean cytokine expression±SD of three biological replicates. Differences between *Aspergillus*-stimulated cells found to be statistically significant by one-way ANOVA with Bonferonni’s Multiple Comparison post-test are indicated (* - p<0.05; ** - p<0.01; *** - p<0.001; **** - p<0.0001). (B) Table summarizing the main observations of cytokine production in response to fungal stimulation as a result of knocking down the stated genes in D1 in comparison with that of control-shRNA transduced control cells. Abbreviations used: Untreated (Unt); *A. fumigatus* swollen conidia (A-sw); non-targeting control shRNA (Ctrl).


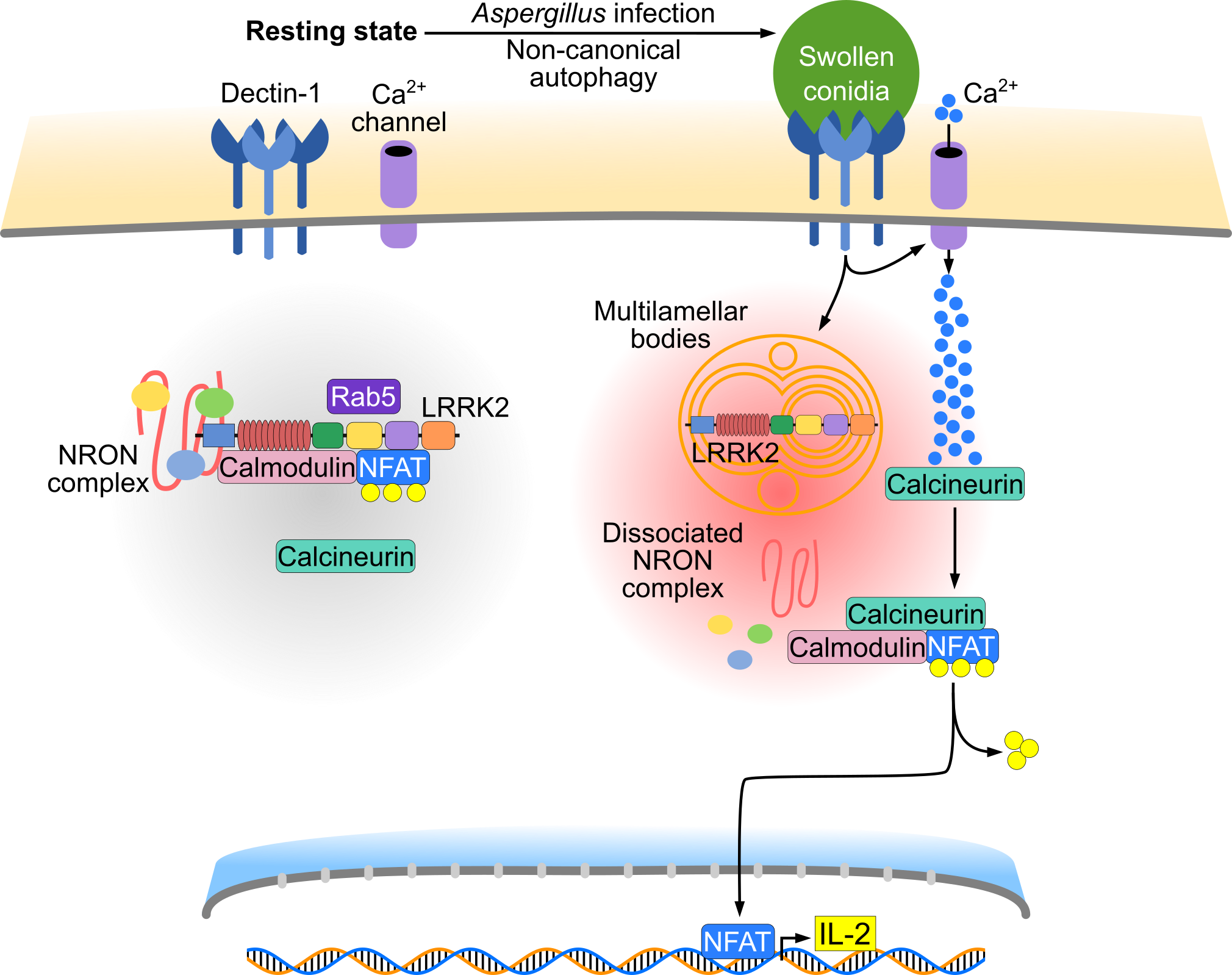


Figure S8: The regulation of the NFAT pathway in DCs is likely accomplished by cellular sequestration and autophagy. At resting state in DCs, LRRK2 and the NRON complex (left panel) are localized in the cytoplasm interacting with NFAT. When *Aspergillus* engages Dectin-1, autophagy and the formation of multilamellar bodies is triggered, sequestering LRRK2, and inducing a disassembly of the NRON complex. Calcineurin, activated by the Ca^2+^ flux triggered as well by Dectin-1 binding to *Aspergillus*, is then able to mediate the dephosphorylation and activation of NFAT (right panel), ultimately transcribing for IL-2.
